# Improved attention-based PCNN with GhostNet for epilepsy seizure detection using EEG and fMRI modalities: extractive pattern and histogram feature set

**DOI:** 10.3389/frai.2025.1679218

**Published:** 2026-01-12

**Authors:** Sunkara Mounika, Reeja S. R.

**Affiliations:** School of Computer Science and Engineering, VIT-AP University, Amaravati, India

**Keywords:** deep learning, EEG, epilepsy seizure detection, fMRI, S-HPCGN

## Abstract

**Introduction:**

Detecting epileptic seizures remains a major challenge in clinical neurology due to the complex, heterogeneous, and non-stationary characteristics of electroencephalogram (EEG) signals. Although recent machine learning (ML) and deep learning (DL) approaches have improved detection performance, most methods still struggle with limited interpretability, inadequate spatial–temporal modeling, and suboptimal generalization. To address these limitations, this study proposes an enhanced hybrid parallel convolutional-GhostNet framework (HPG-ESD) for robust seizure detection using multimodal EEG and functional Magnetic Resonance Imaging (fMRI) data.

**Methods:**

The experimental data consist of pediatric scalp EEG recordings from 24 subjects in the CHB-MIT dataset (22-channel 10–20 system, 256 Hz sampling, continuous multi-hour recordings) and resting-state 3T fMRI scans from 52 participants in the UNAM TLE dataset (26 epilepsy patients and 26 healthy controls). EEG data underwent Gauss-based median filtering, while fMRI images were denoised using an adaptive weight-based Wiener filter. Spatial, temporal, and spectral EEG features were extracted alongside an enhanced common spatial pattern (E-CSP) representation, whereas fMRI features were obtained using deep 3D CNN embeddings combined with a smoothened pyramid histogram of oriented gradients (S-PHOG) descriptor. These multimodal features were fused within a soft voting hybrid parallel convolutional–GhostNet (S-HPCGN) model, integrating an improved attention based parallel convolutional network (IAPCNet) and GhostNet to capture complementary spatial–temporal patterns.

**Results:**

The proposed HPG-ESD framework achieved an accuracy of 0.941, precision of 0.939, and sensitivity of 0.944, outperforming conventional unimodal and state-of-the-art methods.

**Discussion:**

These results demonstrate the potential of multi-modal learning and lightweight attention-enhanced architectures for reliable and clinically relevant seizure detection.

## 1 Introduction

Epilepsy is a chronic neurological disorder characterized by a long-term predisposition to generate epileptic seizures, while a seizure is a transient episode of abnormal, excessive, synchronous neuronal activity in the brain. Although related, these two terms represent distinct clinical concepts that must be clearly differentiated to ensure accurate diagnosis and interpretation of neural abnormalities (Wang et al., 2021).

Epileptic seizures may involve sudden convulsions, sensory disturbances, or brief lapses in consciousness, significantly affecting patients' psychological, cognitive, and emotional wellbeing (Lin et al., 2025). Given the unpredictable nature of epilepsy and its associated comorbidities, including cognitive decline, depression, and psychiatric complications, early and precise detection is essential to minimize long-term health consequences and support effective clinical management (Kok et al., 2022).

Electroencephalography (EEG) remains the primary tool for epilepsy diagnosis due to its ability to capture real-time neural electrophysiological activity in a cost-effective and non-invasive manner (Tsai et al., 2023). However, the manual review of long-term EEG recordings is labor-intensive, subjective, and prone to human error, highlighting the need for automated seizure detection systems that can provide rapid and reliable clinical support (Guo et al., 2022). Brain–computer interface (BCI) technologies further enhance the interaction between neural activity and computational systems, enabling both non-invasive and invasive acquisition of brain signals to facilitate neurorehabilitation, cognitive assessment, and abnormal activity suppression (Li et al., 2021; Kamakshi and Rengaraj, 2024; Yan et al., 2022; Boonyakitanont and Songsiri, 2021). In parallel, multimodal imaging—particularly the integration of EEG and functional MRI—combined with high-performance computing has accelerated biomedical research and improved understanding of seizure dynamics (Sabor et al., 2022).

Beyond the emergence of machine learning (ML) and deep learning (DL), epilepsy research has historically relied on a wide range of signal-processing techniques designed to capture temporal, spectral, and spatial abnormalities in EEG and functional magnetic resonance imaging (fMRI) data. Classical approaches such as autoregressive (AR) modeling, power spectral density (PSD) estimation, coherence analysis, wavelet transforms, and time–frequency decomposition have demonstrated strong diagnostic value by quantifying rhythmicity, frequency shifts, and connectivity disruptions characteristic of seizure activity. Prior studies have explored optimal AR model order selection for seizure detection, coherence predictors for intracortical EEG analysis, and comprehensive surveys of feature extraction techniques for epileptic seizure identification, further establishing the relevance of handcrafted features in understanding epileptogenic patterns (Farooq et al., 2023; Tran et al., 2022; Liu et al., 2023).

With rapid advances in computational intelligence, ML and DL have become essential tools for seizure detection and classification, improving the automation and accuracy of epilepsy diagnosis (Baghersalimi et al., 2021). Various models, including logistic regression (LR), naive Bayes (NB), random forest (RF), linear discriminant analysis (LDA), support vector machines (SVM), recurrent neural networks (RNN), and convolutional neural networks (CNN), have been successfully applied to differentiate seizure types and detect abnormal brain states from EEG data (Gramacki and Gramacki, 2022). Despite their success, many existing approaches still face limitations such as overfitting, insufficient generalization, and high computational complexity, which ultimately restrict their clinical applicability (Hassan et al., 2022). These challenges underscore the need for more robust feature extraction strategies, advanced multimodal integration, and efficient model architectures capable of achieving high diagnostic performance with reduced complexity (Zeng et al., 2023).

The integration of EEG and fMRI enables a richer spatio-temporal characterization of seizure activity, addressing the limited spatial resolution seen in EEG-only systems. The G-MF and AW-WF preprocessing modules enhance data quality by adaptively suppressing mixed noise while preserving critical structural and temporal details that conventional filters typically distort. The proposed feature extraction pipeline, which incorporates E-CSP and S-PHOG, generates more stable and noise-resistant representations through adaptive frequency weighting and smoothing strategies. Additionally, the hybrid S-HPCGN architecture combines attention-enhanced feature learning in IAPCNet with the lightweight yet expressive feature generation of GhostNet, providing an improved balance between computational efficiency and discriminative capability. The soft voting strategy further strengthens the system by merging complementary confidence scores from multimodal learners, resulting in more reliable and consistent seizure detection. These design characteristics represent significant methodological advancements that extend beyond numerical performance improvements.

While the individual components of the proposed framework build upon established concepts, the overall architecture introduces a unified theoretical design that is structurally distinct from existing SOTA approaches. The HPG-ESD model is formulated around a coordinated multimodal learning principle, where EEG-derived temporal activations and fMRI-derived spatial signatures are projected into a shared latent domain through parallel, attention-regulated pathways. This coupling is absent in conventional models, which typically treat the modalities independently or fuse them at a surface-level feature concatenation.

The internal interaction between IAPCNet and GhostNet forms a heterogeneous learning mechanism in which high-capacity attention-driven features and lightweight intrinsic features reinforce each other through probability-aligned soft fusion. This creates an adaptive cross-modal consistency that cannot be achieved by simply stacking existing models. The resulting architecture therefore represents a structurally integrated system governed by a specific multimodal learning theory, rather than a collection of incremental improvements.

The main contributions of this work are as follows:

- Proposing a G-MF and AW-WF-based preprocessing technique for improving the quality of WWG signals and fMRI images, respectively. These approaches adopt a Gaussian filter and a hybrid adaptive weighting function to preserve sharp transitions and enhance performance.
- Extracting E-CSP and S-PHOG-based features that adopt an activation function with weighted frequencies and Gaussian smoothing to avoid overfitting and sensitivity to minor pixel variation.
- Contributing the S-HPCGN model that integrates IAPCNet and GhostNet models. Each of these models trains the extracted features and provides prediction scores to compute the soft voting policy. This approach yields good detection results with higher probability classes.

The rest of the paper is organized as follows: Section 2 outlines challenges in existing epileptic seizure detection methods. Section 3 introduces the proposed multimodal EEG-fMRI approach. Section 4 compares its performance with existing methods, and Section 5 concludes the study.

## 2 Literature review

This section reviews a wide range of techniques for epileptic seizure detection, focusing on advanced methods while also identifying the potential of multimodal approaches like EEG-fMRI integration to enhance accuracy and interpretability.

In 2023, Mohammad and Al-Ahmadi focused on epileptic seizure detection using a multi-source dataset of EEG signals and brain MRIs (Mohammad and Al-Ahmadi 2023). Feature extraction is performed via two parallel streams: SVD-Entropy and wavelet transform for EEG, as well as CNN for MRI. Moreover, the retrieved features are subsequently fused into a single vector and classified using an SVM to identify epileptic seizures.

In 2024, Tang et al. proposed an automatic epilepsy detection framework leveraging path signature features and a Bi-LSTM network with attention (Tang et al., 2024). The path signature extracts discriminative features capturing dynamic channel dependencies in EEG, while the Bi-LSTM with attention models temporal dependencies. The method was tested on public datasets, along with a private hospital dataset, using leave-one-out and five-fold cross-validation.

In 2025, Sikarwar et al. introduced an automatic epilepsy detection approach using EEG signals, combining advanced entropy measures with modern preprocessing methods (Sikarwar et al., 2025). EEG signals were denoised using adaptive wavelet models to preserve their integrity. Features extracted include mvMPE and mvMFE to characterize complexity and frequency variations. UMAP was applied for non-linear dimensionality reduction to enhance feature discrimination. The model employed a ResNet integrated with Bi-LSTM to capture both temporal and spatial information.

In 2022, Yuan et al. proposed a method for automatic epileptic seizure detection based on kernel-driven robust ProCRC combined with GNMF (Yuan et al., 2022). Wavelet transform was first used to preprocess raw EEG signals to derive time–frequency distributions as initial features. GNMF reduces dimensionality while preserving important EEG characteristics. Subsequently, the robust ProCRC method classifies test samples by maximizing the likelihood of their belonging to seizure or non-seizure classes.

In 2022, Song et al. proposed a single-channel seizure detection framework based on BRRM and an optimized model named ONASNet (Song et al., 2022). BRRM visualizes how brain rhythms repeat over time by mapping them in phase space, revealing the underlying non-linear characteristics of EEG activity. Furthermore, transfer learning was employed to apply ONASNet to the EEG dataset. Together, BRRM and ONASNet enable the simultaneous extraction of features from various brain rhythms by utilizing multiple neural network channels.

In 2024, Sadiq et al. proposed a Hellinger distance classifier combined with PSO to improve feature selection in EEG signals (Sadiq et al., 2024). This approach enhances classification accuracy and reduces the time and dimensionality of the dataset. Their findings highlight the method's effectiveness for academic and clinical use, offering precise detection of epileptic seizures.

In 2023, Prasanna et al. presented BESD-Net, a deep learning framework incorporating recurrent learning for seizure detection (Prasanna et al., 2023). The initial step involved preprocessing the EEG data to eliminate irrelevant noise. A specialized CCNN was trained on this preprocessed dataset to accurately extract features correlated with epilepsy. Additionally, these features were optimized using ERF-based feature selection, which prioritized those with strong relevance to the disease.

In 2010, Aydin presented a step-wise least squares estimation algorithm (SLSA), implemented in the Matlab ARfit package, to clinical EEG data for accurate estimation of auto-regressive (AR) model orders for both normal and ictal signals, with PSD derived using the Burg method (Aydin, 2010). They reported that ARfit was more useful than traditional criteria such as FPE, AIC, MDL, and CAT for EEG discrimination. Overall, they concluded that SLSA was superior due to its non-heuristic nature, lower computational complexity, and ability to generate more reliable AR order estimates for long EEG sequences.

In 2009, Aydin presented a comparative study of two auto-regressive (AR) methods (Burg and Yule–Walker) and two subspace-based techniques (Eigen and MUSIC) for power spectral density estimation in computing the coherence function (CF) to assess EEG synchronization between hemispheres (Aydin, 2009). Using intracortical EEG from WAG/Rij rats, they found that AR-based methods produced similar outcomes but were highly sensitive to model order, while subspace methods detected specific CF peaks but required higher computational complexity. They concluded that high-order Burg modeling was most suitable for EEG synchronization analysis.

In 2023, Ein Shoka et al. presented a comprehensive review of epilepsy, describing it as a central nervous system disorder characterized by abnormal brain activity and recurrent seizures (Ein Shoka et al., 2023). They highlighted the heavy reliance on EEG signals for seizure analysis and noted that manual seizure identification was time-consuming. Their work summarized preprocessing steps, feature extraction, and classification methods while also outlining methodological limitations, challenges, and future research directions in automated EEG-based seizure detection.

Table 1 summarizes recent studies on epileptic seizure detection, emphasizing the approaches used along with their benefits and limitations.

**Table 1:** Review of existing works.

**Author [Citation]**	**Methods**	**Benefits**	**Limitations**
Mohammad and Al-Ahmadi (2023)	CNN, SVM	It effectively manages large feature spaces and provides robust generalization across various datasets	Should implement advanced similarity metrics to further enhance epileptic seizure detection
Tang et al. (2024)	Bi-LSTM	It validates that the proposed approach generalizes effectively	Attention must be given to enlarging the dataset for optimal performance
Sikarwar et al. (2025)	ResNet-Bi-LSTM	Using UMAP for dimensionality reduction increases the features' effectiveness in distinguishing classes	Developing compact models suited for real-time operation will support uninterrupted monitoring with wearable devices
Yuan et al. (2022)	ProCRC	It strengthens class separability and accurately predicts the most likely class label for test EEG samples	Subsequent studies should investigate boosting the algorithm's accuracy with a reduced amount of labeled data
Song et al. (2022)	ONASNet	The method effectively represents both seizure and seizure-free signals while clearly separating them from healthy patterns	Exploring these algorithms and scaling our method from single-channel to multi-channel EEG signals is a worthwhile direction for future work
Sadiq et al. (2024)	Hellinger distance classifier with PSO	It reduces the impact of imbalanced data and effectively manages high-dimensional datasets by detecting closely related numerical objects	Additional research is needed on integrating the Hellinger distance classifier with deep learning methods
Prasanna et al. (2023)	CCNN	The model outperforms other methods by delivering improved accuracy and reduced loss	Incorporating cutting-edge deep learning approaches and investigating transfer learning will improve how well the model generalizes

### 2.1 Problem statement

Detecting epileptic seizures from EEG signals is challenging due to their inherent complexity, high dimensionality, noise, and variations between individuals. Effective seizure prediction requires robust preprocessing, discriminative feature extraction, and accurate classification techniques. Recent studies have explored various ML and DL models to improve detection performance. Mohammad and Al-Ahmadi (2023) employed CNN and SVM, which handle large feature spaces well and generalize across datasets, although performance could benefit from more advanced similarity metrics. Tang et al. (2024) demonstrated that Bi-LSTM can generalize effectively but stressed the need for larger datasets. Similarly, Sikarwar et al. (2025) improved class separability using ResNet-Bi-LSTM with UMAP for dimensionality reduction, although more compact models are needed for real-time applications. Meanwhile, Prasanna et al. (2023) showed that their CCNN-based BESD-Net model achieved high accuracy, suggesting that further exploration of advanced deep learning and transfer learning techniques could improve generalization. Despite these advancements, relying solely on EEG limits the understanding of underlying neural mechanisms. EEG captures electrical activity but lacks spatial resolution, restricting the localization of seizure onset zones. Hence, there is a growing need to incorporate multimodal neuroimaging data, such as functional magnetic resonance imaging (fMRI), which provides complementary spatial information about brain activity. The integration of EEG with fMRI can enhance the interpretability of features and improve classification by capturing both temporal and spatial dynamics of seizures. This multimodal approach can enable more accurate, personalized, and clinically relevant seizure detection systems.

Recent studies have emphasized the importance of temporal–frequency attention for EEG and multimodal neuroimaging analysis. Methods such as Fourier attention (Ke et al., 2024) and wavelet-based attention mechanisms (Wang et al., 2025a,c) dynamically emphasize discriminative frequency components, enabling more precise modeling of seizure-related oscillations. These attention mechanisms operate by adaptively weighting temporal and spectral representations, which aligns conceptually with the non-linear, frequency-aware design of the proposed E-CSP and S-PHOG modules. Additionally, hybrid frameworks that combine attention mechanisms with non-linear feature extraction, such as semantic-aware fusion models and deep neurodynamic attention networks (Wang et al., 2025b; Ke et al., 2023), demonstrate the growing significance of integrating attention with advanced feature transformation. Incorporating these theoretical perspectives broadens the methodological context of the proposed HPG-ESD model and highlights the relevance of multimodal spatiotemporal learning in epilepsy detection.

## 3 Proposed methodology

Epilepsy is a persistent brain disorder characterized by spontaneous seizures triggered by irregular electrical activity in the brain. Detecting these seizures promptly and accurately is essential for proper diagnosis, treatment, and ongoing patient care. While EEG is widely used for seizure detection due to its excellent temporal resolution, it often struggles to precisely identify the seizure origin because of its limited spatial detail. Functional magnetic resonance imaging (fMRI), on the other hand, provides high-resolution spatial mapping of brain function through hemodynamic signals. Integrating EEG with fMRI offers a powerful method to enhance seizure detection by combining EEG's rapid temporal data with the detailed spatial insights of fMRI. This multimodal strategy improves the precision and reliability of detecting and localizing seizures, thereby supporting more effective clinical management.

A deeper understanding of the fusion mechanism reveals how EEG and fMRI together enhance seizure detection in ways that a single modality cannot. EEG contributes rich temporal cues reflecting abrupt neuronal discharges, while fMRI provides detailed spatial information about the distribution of abnormal hemodynamic responses. The proposed architecture aligns these fast temporal patterns with spatial activation maps, enabling the model to learn cross-modal correspondences that accurately localize and characterize seizure activity. This complementary interaction forms the basis for the improved robustness and precision of the multimodal HPG-ESD framework. This study proposes a novel hybrid parallel convolutional-GhostNet model for epilepsy seizure detection (HPG-ESD).

**Figure 1:**
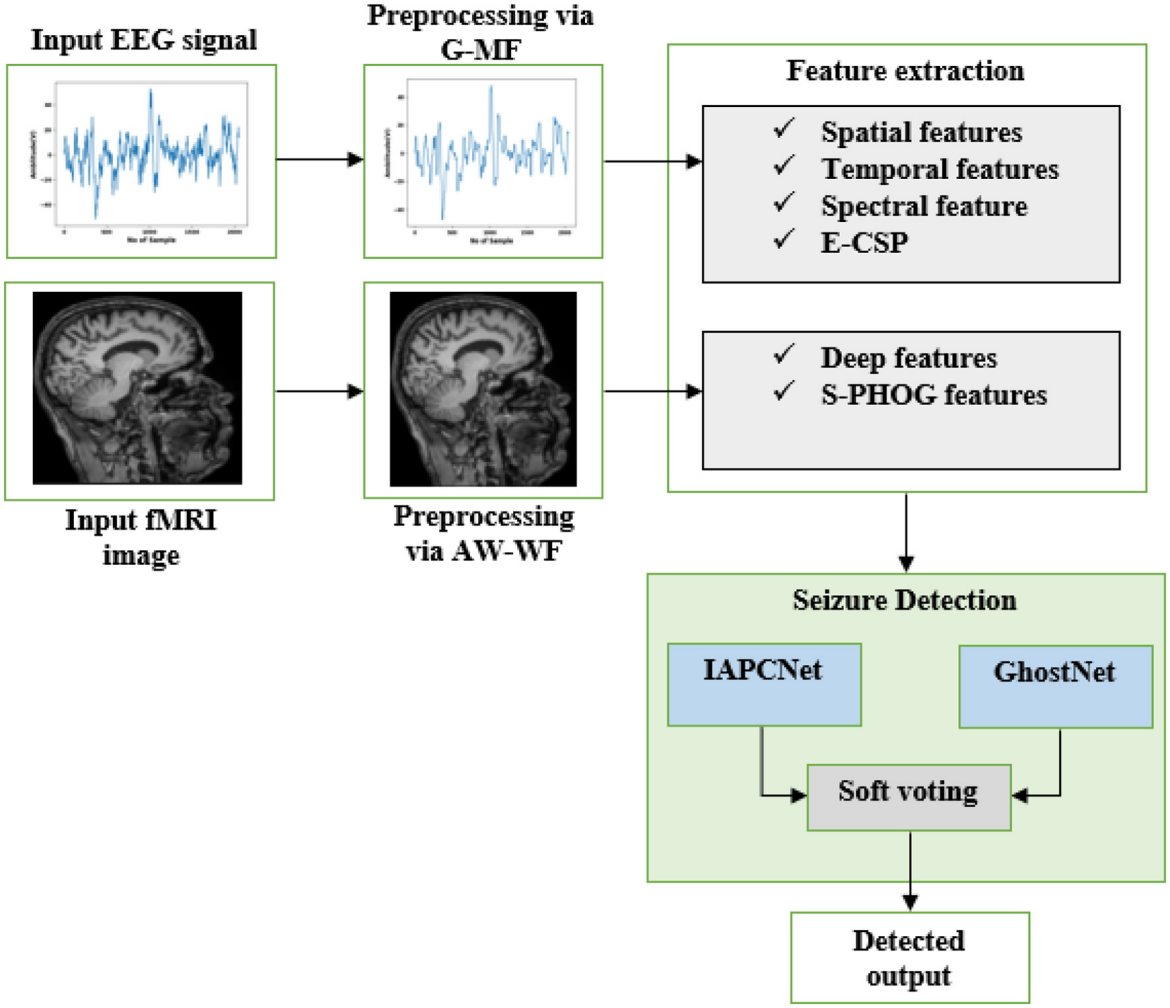
Outline of proposed HPG-ESD model.

As shown in Figure 1, the seizure detection process begins with the acquisition of two types of input data: EEG signals and fMRI images. Each modality undergoes preprocessing to enhance signal clarity and suppress unwanted noise; EEG signals are filtered using a Gauss-based median filter (G-MF), while fMRI images are denoised with an adaptive weight-based Wiener filter (AW-WF). Next, important features are extracted from each modality to capture relevant seizure-related information. For EEG signals, spatial, temporal, and spectral features are derived, along with an enhanced common spatial pattern (E-CSP) technique to better discriminate seizure activity.

For fMRI images, deep features are extracted alongside an S-PHOG descriptor to effectively represent spatial patterns. The processed features from both EEG and fMRI are then fed into an S-HPCGN model that combines IAPCNet and GhostNet architectures, which collaboratively detect seizure events by leveraging their complementary strengths. Finally, a soft voting approach aggregates the predictions from the hybrid models to generate a more robust and accurate final seizure detection output.

The design of the HPG-ESD architecture is grounded in the need for a unified representation that retains fine-grained temporal information from EEG signals while capturing spatially distributed neurovascular patterns in fMRI data. The dual-branch structure of IAPCNet enables simultaneous learning of complementary feature spaces, where depth-wise separable convolution and adaptive normalization enhance stability and reduce redundancy. The spatial–perceptual attention mechanism introduces anisotropic weighting across the feature maps, improving sensitivity to seizure-related activations that may manifest differently across modalities. The parallel integration of IAPCNet with GhostNet forms a heterogeneous learner ensemble, in which lightweight intrinsic feature generation complements high-capacity attention-enhanced encoding. The soft voting formulation extends classical ensemble averaging by incorporating calibrated probability distributions derived from both deep features and frequency-weighted handcrafted descriptors, allowing proportional influence based on modality reliability. This design establishes a coherent theoretical basis for multimodal fusion, reducing overfitting and supporting improved discriminative separation between seizure and non-seizure patterns.

### 3.1 Preprocessing

The initial phase of seizure detection involves preprocessing, which focuses on refining the data by removing noise and artifacts to ensure more accurate feature extraction and classification. Let us assume that *I*^*sig*^ is the input EEG signal and *I*^*img*^ is the input fMRI image, which are preprocessed using an enhanced approach discussed as follows:

#### 3.1.1 EEG preprocessing

EEG recordings frequently include various types of noise, such as muscle artifacts, eye blinks, and electrical interference. To mitigate these, an enhanced median filter is used. This filter excels at eliminating impulsive noise while preserving vital signal details, particularly abrupt changes associated with seizures. By smoothing out unnecessary fluctuations, the Gauss-based median filter (G-MF) boosts the signal-to-noise ratio (SNR) and maintains essential EEG features for accurate seizure detection. The Gauss-based Median Filter (G-MF) extends median filtering (MF) (Song and Liu, 2019), which is a non-linear technique used to reduce noise in signals*I*^*sig*^. It operates by taking a sliding window of neighboring values from the signal, sorting them based on magnitude, and replacing the center value with the median of the sorted values. The standard form of MF is formulated as in Equation 1.


$$Mf=Med\left\{I^{sig}\left(t-k\right),I^{sig}\left(t+k\right)\right\},$$ (1)

for k = −N to N

where *I*^*sig*^(*t*) denotes the input signal and *k* represents the index of window size.

The main limitation of MF is its inefficiency in variable-noise environments, leading to poor performance. To address this limitation, the G-MF technique is proposed, adopting a Gaussian filter approach. The process of G-MF is as follows:

**Step 1**: apply the Gaussian filter as in Equation 2.


$$\begin{array}{l} y^{\prime }\left(t\right)=\sum_{k=-N}^{N}g\left(k\right).I^{sig}\left(t+k\right) \end{array}$$ (2)

where *g*(*k*) denotes the Gaussian kernel, defined as in Equation 3. Here, σ is estimated using Equation 4, var_*i*_ denotes the local variance of the signal point *i*, and φ represents a tunable parameter (0, 1).


$$\begin{array}{l} g\left(k\right)=\frac{1}{\sqrt{2\pi \sigma^{2}}}.e^{-k^{2}/2\sigma^{2}} \end{array}$$ (3)

$$\begin{array}{l} \sigma_{i}=\varphi .\sqrt{{\mathrm{var}}_{i}} \end{array}$$ (4)

**Step 2**: apply the median filter to the smoothed output *y*(*t*) to obtain the G-MF signal as in Equation 5.


$$\begin{array}{l} Mf_{new}=Med\left\{y^{\prime }\left(t-k\right),y^{\prime }\left(t+k\right)\right\}, \end{array}$$ (5)

for k = −N to N

where *y*′(*t*) refers to the output of the Gaussian filter, *N* denotes the window size, and the filtered signal is denoted as *Mf*_*new*_.

#### 3.1.2 fMRI preprocessing

Functional MRI (fMRI) image data often contains noise and distortions caused by scanner errors, patient movement, and physiological variations. To address this, an adaptive weight-based Wiener filter (AW-WF) is applied during preprocessing. The AW-WF is a variant of the Wiener filtering (Kalaivani and Phamila, 2018). The traditional formulation of WF is defined as in Equation 6.


$$\begin{array}{l} Wf\left(n,m\right)=M+\left(\frac{\sigma^{2}-v^{2}}{\sigma^{2}}\right).\left(I^{img}\left(n,m\right)-M\right) \end{array}$$ (6)

where *M* denotes mean, σ^2^ denotes variance, *v*^2^ indicates noise variance of the mask matrix, and *I*^*img*^(*n, m*) indicates the noisy image.

This WF filter adjusts based on the local image variance to effectively reduce random noise while preserving sharp edges and critical spatial information. However, the WF performs poorly for noisy images. Adopting mean and variance functions can lead to edge blurring problems. To address this issue, the AW-WF procedure is introduced, which employs a hybrid adaptive weighting function. The procedure to be followed for AW-WF is as follows:

Step 1: determine the PSD by performing a Fourier transform on the image's autocorrelation function, for both the noisy and the original images.

Step 2: place a filtering mask centered over a specific pixel in the noisy image.

Step 3: collect and organize the intensity values of all pixels located within the area enclosed by the mask.

Step 4: calculate the average (mean) of these intensity values and allot it to the central pixel of the mask.

Step 5: evaluate the local average (mean, μ) and the local variance (σ^2^) within the region covered by the mask.

Step 6: estimate the new pixel value *Wf*_*new*_(*n, m*) as in Equation 7.


$$\begin{array}{l} Wf_{new}\left(n,m\right)=\beta .Med+\left(1-\beta \right)\\ .\left[M+\left(\frac{\sigma^{2}-v^{2}}{\sigma^{2}}\right).\left(I^{img}\left(n,m\right)-M\right)\right] \end{array}$$ (7)

where *Med* indicates the median value of the local window, *M* refers to the mean as defined in Equation 8, σ^2^ denotes variance as defined in Equation 9, *v*^2^ indicates noise variance of the mask matrix, *I*^*img*^(*n, m*) indicates the noisy image, and β indicates a tunable parameter, which is computed using the “hybrid adaptive weighting function” as in Equation 10.


$$\begin{array}{l} M=\frac{1}{nm}\sum_{n,m\in I^{img}}I^{img}\left(n,m\right) \end{array}$$ (8)

$$\begin{array}{l} \sigma^{2}=\frac{1}{nm}\sum_{n,m\in I^{img}}{I^{img}}^{2}\left(n,m\right)-M^{2} \end{array}$$ (9)

$$\begin{array}{l} \beta =\frac{v^{2}}{\sigma^{2}+\lambda .\alpha \left(m,n\right)+\varepsilon } \end{array}$$ (10)

where *v*^2^ indicates noise variance (global), σ^2^ denotes local variance, ε specifies a small constant to avoid division by zero, λ denotes the sensitivity parameter (0, 1), and $\alpha \left(m,n\right)=\sqrt{B_{x}^{2}+B_{y}^{2}}$, which is the gradient magnitude using Sobel. Here, $B_{x}^{2}$ and $B_{y}^{2}$ are parameters with respect to axes x and y, respectively.

Step 7: repeat the step 2 to step 6 for every pixel in the noisy image.

Thus, the AW-WF technique provides a filtered image *Wf*_*new*_ that better handles the mixed noise of median filtering with the Wiener filter. Using an adaptive weighting function helps preserve strong edges while effectively reducing noise, leading to improved performance.

### 3.2 Feature extraction

Extracting features is an important phase where significant insights are derived from preprocessed EEG and fMRI (*Mf*_*new*_ and *Wf*_*new*_) to support accurate seizure identification. This step involves converting complex, multidimensional data into a set of helpful features that reveal underlying seizure-related patterns.

The CHB-MIT EEG recordings consist of continuous multi-hour scalp recordings sampled at 256 Hz, with individual EDF files typically containing uninterrupted 1-h segments. All recordings follow the international 10–20 electrode placement system, comprising 22–24 scalp electrodes positioned across frontal, temporal, central, parietal, and occipital regions (e.g., Fp1/Fp2, F3/F4, C3/C4, T3/T7, T4/T8, P3/P4, O1/O2, Cz, Pz). After preprocessing, the continuous EEG signals are directly used for feature extraction without temporal segmentation. Temporal features (e.g., Hjorth parameters, line length, zero-crossing rate) and spectral features are computed per electrode using Welch's PSD method, capturing band-specific activity in delta, theta, alpha, beta, and gamma ranges. Electrode-wise features are then concatenated in a fixed 10–20 order, and spatial structure is modeled using enhanced common spatial patterns (E-CSP) to exploit inter-electrode covariance relationships and highlight focal seizure activity. The resulting temporal, spectral, and spatial descriptors are combined to form the final EEG feature vector for each recording.

#### 3.2.1 Extraction of EEG-based features

EEG signals provide valuable information about brain activity through electrical impulses recorded over time. Extracting meaningful features from the preprocessed signals *Mf*_*new*_ is essential for accurately detecting epileptic seizures. The extraction process focuses on capturing different aspects of the EEG that reflect seizure-related changes in brain function.

##### 3.2.1.1 Spatial features

Spatial features (Zeng et al., 2021) from EEG signals capture the distribution and interaction of electrical activity across different regions of the brain. Since seizures often originate in specific areas and can spread to neighboring regions, analyzing the spatial patterns of EEG channels helps identify the location and extent of abnormal brain activity. For this purpose, CNN-based spatial features are extracted *S*_1_. These features reflect how signals from different electrodes relate to each other in space, providing insights into the spatial organization of seizure events. By examining these spatial dynamics, seizure-related patterns can be more accurately detected and localized.

##### 3.2.1.2 Temporal features

Temporal features (Zeng et al., 2021) describe how EEG signals change over time, capturing the dynamic behavior of brain activity. Since epileptic seizures are characterized by sudden and irregular changes in signal patterns, analyzing temporal aspects such as signal amplitude, duration, variance, and waveform shape can provide crucial information. To achieve this, Bi-LSTM-based temporal features are extracted as *S*_2_. These features help identify transient events, rhythmic discharges, or spikes that occur during seizure episodes. By studying the time-domain characteristics of the EEG, it becomes possible to detect the progression, onset, and termination of seizures more accurately.

##### 3.2.1.3 Spectral features

Spectral roll-off (Chandwadkar and Sutaone, 2012) refers to the frequency below which a fixed percentage of the total spectral power lies. This feature *S*_3_is commonly used to understand the imbalance or tilt in the frequency content of a signal window.

##### 3.2.1.4 Enhanced common spatial pattern (E-CSP)

Common spatial pattern (CSP) (Jiang et al., 2018) is a supervised technique designed to identify spatial filters that enhance variance for one class while simultaneously reducing it for the opposite class. It uses the average concentration level as a reference baseline to better capture task-related changes. It involves centering the task-related signal $Z_{i}^{task}$ by subtracting the average of the initial-state signal$Z_{i}^{task}$, resulting in a new signal ${\overset{▪}{Z}}_{i}^{task}$ as defined in Equation 11.


$$\begin{array}{l} {\overset{▪}{Z}}_{i}^{task}=sigmoid\left(Z_{i}^{task}\right)=\frac{1}{1+e^{-Z_{i}^{task}}} \end{array}$$ (11)

This adjustment emphasizes changes in concentration after task onset. The centered signal is then passed through a sigmoid function, compressing its range into [0, 1] for normalization and stability, making the features more robust for classification. In E-CSP, this non-linear activation helps reduce the effect of noise or outliers and ensures the features are bounded and stable for learning. All frequency components are considered equally, without frequency weighting, which may introduce noise or irrelevant information. To address this, an enhanced common spatial pattern (E-CSP) is suggested that adjusts the activation function. The formulation of E-CSP is defined as in Equation 12.


(12) $$\overset{\cdot \cdot }{Z}i^{task}=swish(\overset{\cdot }{Z}i^{task})$$ (12)

where ${\overset{▪}{Z}}_{i}^{task}=softsign\left({\overset{\wedge }{Z}}_{i}^{task}\right)$ which means ${\overset{▪}{Z}}_{i}^{task}=\frac{{\overset{\wedge }{Z}}_{i}^{task}}{1+|{\overset{\wedge }{Z}}_{i}^{task}|}$. Here, ${\overset{\wedge }{Z}}_{i}^{task}$ is the normalized signal by subtracting the average of the resting-state signal (RSS), and it is defined as ${\overset{\wedge }{Z}}_{i}^{task}=\frac{Z_{i}^{task}-\mu^{Rs}}{\sigma^{Rs}}$. The mean of the RSS is $\mu^{Rs}=\frac{1}{N}\sum_{i=1}^{N}z_{i}^{Rs}$ and the standard deviation of the RSS is $\sigma^{Rs}=\sqrt{\frac{1}{N}\sum_{i=1}^{N}{\left(z_{i}^{Rs}-\mu^{Rs}\right)}^{2}}$. Next, $\overset{\cdot \cdot }{Z}i^{task}$ is used as the input to the objective function of the E-CSP algorithm, which seeks to maximize the ratio of the squared averages between two signal sets. The conventional formulation is given below in Equation 13.


H2(Z,ω)=1n1 ∑i∈k1‖ωTZ··itask‖21n2 ∑i∈k2‖ωTZ··itask‖2                    =1n1 ∑i∈k1ωTGiω1n2 ∑i∈k2ωTGiω=ωTG¯1ωωTG¯2ω (13)

To obtain robustness and training stability, Equation 13 is improved as in Equation 14.


$$\begin{array}{l} {H_{2}}^{new}\left(Z,\omega \right)=\frac{\omega^{T}{{Ḡ}_{1}}^{\prime }\omega }{\omega^{T}{{Ḡ}_{2}}^{\prime }\omega } \end{array}$$ (14)

where ${H_{2}}^{new}\left(Z,\omega \right)$ is the objective function, Z illustrates the input data, ω denotes the optimal spatial filter, ω^*T*^represents the transpose of ω, ${{Ḡ}_{1}}^{\prime }$ and ${{Ḡ}_{2}}^{\prime }$ signify the covariance matrix for the task and resting states and are defined as given in Equations 15, 16, respectively:


$$\begin{array}{l} {{Ḡ}_{1}}^{\prime }=\left(1-\gamma_{1}\right){Ḡ}_{1}+\gamma_{1}J \end{array}$$ (15)

$$\begin{array}{l} {{Ḡ}_{2}}^{\prime }=\left(1-\gamma_{2}\right){Ḡ}_{2}+\gamma_{2}J \end{array}$$ (16)

where *n*_1_ denotes the count of task class, *k*_1_ the refers to task signals, *J* the denotes identity matrix of Ḡ_1_and Ḡ_2_shape, respectively, and *W*_*freq*_(*f*) denotes the frequency weighting function as defined in Equation 17. Here, *f* denotes frequency, Δ*f* indicates bandwidth, *f*_*c*_ denotes center frequency of the band, andγ_1_, γ_2_ denotes the tuning parameter.


$$\begin{array}{l} W_{freq}\left(f\right)=\frac{1}{1+4{\left(\frac{f-f_{c}}{\Delta f}\right)}^{2}} \end{array}$$ (17)

Additionally, ${\bar{G}}_{1}=\frac{1}{n_{1}}\underset{i=k}{\sum }(W_{freq}(f).\overset{\cdot \cdot }{Z}i^{task}\overset{\cdot \cdot }{Z}i^{task})$. Here, *n*_2_ denotes the count of the resting state class and *k*_2_ refers to resting-state signals.

The filter is derived using singular value decomposition, as shown in Equations 18, 19 represents the transformation of $\overset{\cdot \cdot }{Z}i^{task}into\;y_{i}^{j}$.


$$({{\bar{G}}_{2}}^{'-1}-{\bar{G}}_{1})\omega_{j}={\text{λ}}_{j}\omega_{j}$$ (18)

$$({\bar{G}}_{2}-{\bar{G}}_{1})\omega_{j}={\text{λ}}_{j}\omega_{j}$$ (19)

Finally, the mean of each filtered signal is extracted as *S*_4_. Thus, the extraction of signal-based features is collectively represented as *SF* = [*S*_1_, *S*_2_, *S*_3_, *S*_4_].

#### 3.2.2 Extraction of fMRI-based features

Feature extraction from preprocessed fMRI images *Wf*_*new*_ involves identifying patterns in brain activity that are spatially distributed and relevant to seizure events. Unlike EEG, which captures electrical activity over time, fMRI measures changes in blood flow (hemodynamic responses), providing high-resolution spatial information about which brain areas are active. The extraction process focuses on deep features and PHOG features to recognize seizure-related changes in brain function.

##### 3.2.2.1 Deep features

Deep learning techniques are commonly employed to automatically learn hierarchical features *I*_1_ from preprocessed image*Wf*_*new*_. These deep features uncover intricate and abstract spatial patterns that traditional methods may overlook, aiding in the identification of brain regions involved in seizure activity.

ResNet: Residual network (ResNet) (Liang, 2020) is a deep convolutional neural network that uses residual, or “skip,” connections to address the vanishing gradient issue common in very deep models. These connections help the network learn identity functions, facilitating the training of much deeper architectures. ResNet extracts features hierarchically, beginning with basic spatial elements like edges and textures in the early layers, and advancing to more abstract, high-level representations in deeper layers. These extracted features are valuable for detecting intricate spatial abnormalities in fMRI scans associated with seizures.

VGG16: the VGG16 architecture (Tammina, 2019) consists of 16 layers and is recognized for its clean and uniform structure using small 3 × 3 convolution filters. This simplicity allows it to effectively learn detailed spatial features. When used for feature extraction, VGG16 captures hierarchical representations from basic textures to complex shapes and areas of interest. These features are valuable for identifying abnormal spatial patterns in fMRI scans linked to seizures.

##### 3.2.2.2 Smoothened pyramid histogram of oriented gradients (S-PHOG)

Pyramid histogram of oriented gradients (PHOG) (Saïdani and Kacem Echi, 2014) augments HOG features with spatial pyramid matching to encode shape and spatial structure. The preprocessed image *Wf*_*new*_ is hierarchically partitioned into finer grids, doubling splits per axis at each level, with gradient data in each region forming the pyramid. However, gradient computation is highly susceptible to noise, which can significantly distort both gradient magnitude and orientation. This, in turn, degrades feature quality, resulting in reduced robustness and overall performance. To overcome this limitation, the smoothened pyramid histogram of oriented gradients (S-PHOG) is proposed. In the proposed method, smoothing is implicitly incorporated prior to gradient computation to minimize the effect of noise and improve gradient consistency. This helps enhance the stability and reliability of the orientation features extracted in subsequent stages. The S-PHOG process includes the following steps to obtain the normalized final S-PHOG descriptor *I*_2_.

Step 1-Proposed gradient computation: conventionally, this method employs one-dimensional centered discrete derivative masks in both vertical and horizontal directions. These masks are used to filter the grayscale image, as illustrated in Equation 20. Here, *d*_*x*_and *d*_*y*_represents *x* and *y*derivatives of image *Wf*_*new*_using a convolution operation, accordingly. The gradient of *A*_*x*_and *A*_*y*_is formulated as in Equations 21, 22, accordingly.


$$\begin{array}{l} d_{x}=\left[\begin{array}{ccc} -1 & 0 & 1 \end{array}\right]\;\&\;d_{y}=\left[\begin{array}{c} 1\\ 0\\ -1 \end{array}\right] \end{array}$$ (20)

$$\begin{array}{l} A_{x}=Wf_{new}*d_{x} \end{array}$$ (21)

$$\begin{array}{l} A_{y}=Wf_{new}*d_{y} \end{array}$$ (22)

The method mainly captures edge and gradient details, which limits its ability to represent complex textures or patterns. As a result, it may miss important features relevant to certain tasks. To avoid this problem, the gradient formulation needs to be updated as in Equations 23, 24, respectively.


$$\begin{array}{l} {A_{x}}^{Imp}={\left(Wf_{new}\right)}_{smooth}\times d_{x} \end{array}$$ (23)

$$\begin{array}{l} {A_{y}}^{Imp}={\left(Wf_{new}\right)}_{smooth}\times d_{y} \end{array}$$ (24)

where (*W*_*f*_*new*_)*smooth*_ denotes the Gaussian smoothening image, computed using Equation 25. Here, *G*_σ_denotes the Gaussian kernel with standard deviation σ.


$$\begin{array}{l} {\left(Wf_{new}\right)}_{smooth}=Wf_{new}\times G_{\sigma } \end{array}$$ (25)

Furthermore, the gradient magnitude and orientation are determined as outlined in Equations 26, 27, respectively.


$$\begin{array}{l} A=\sqrt{{A_{x}}^{2}+{A_{y}}^{2}} \end{array}$$ (26)

$$A_{O}=\arctan\frac{A_{y}}{A_{x}}$$ (27)

To obtain stable and reliable gradient computation, the gradient magnitude and orientation formulation are updated as shown in Equations 28, 29, respectively.


$$\begin{array}{l} A_{new}\left(i,j\right)=\frac{A\left(i,j\right)}{\varepsilon_{local}\left(i,j\right)+\varepsilon } \end{array}$$ (28)

Anewo=arctan(υ¯xυ¯y) (29)

where $A\left(i,j\right)=\sqrt{{\left(A_{x}^{imp}\right)}^{2}+{\left(A_{y}^{imp}\right)}^{2}}$, $\varepsilon_{local}\left(i,j\right)=\underset{\left(x,y\right)\in Neigh\left(i,j\right)}{\sum }{\left(A_{x}^{imp}\right)}^{2}+{\left(A_{y}^{imp}\right)}^{2}$, *Neigh*(*i, j*)denotes the local neighborhood around the pixel (*i, j*) and ε indicates a small persistent value to avoid division by zero. Additionally, the average vectors in the neighborhood is ${\bar{\upsilon }}_{x}=mean\left(\upsilon_{x}\right)$ and υ_*x*_ is computed as υ_*x*_ = cos(*A*_*o*_), the average vectors in the neighborhood ${\bar{\upsilon }}_{y}=mean\left(\upsilon_{y}\right)$ and υ_*y*_ is computed as υ_*y*_ = sin(*A*_*o*_).

Step 2: orientation binning builds a histogram per cell by assigning pixel votes to orientation bins according to their gradient directions. If gradients are treated as unsigned, the bins range from 0 to 180°; if signed, the range extends to 360°.

Step 3: the first matrix holds the orientation values assigned to the histogram bins, and the second stores the related gradient magnitudes.

Step 4: HOG features are first extracted over the entire image using Z orientation bins, with each bin counting pixels within a particular angle range. Moreover, the image is then divided into four parts, and HOG descriptors are designed for each. This process is repeated across pyramid levels: level 0 yields a Z-vector, and Level 1 yields a Z-vector. The S-PHOG descriptor is formed by merging histograms from each pyramid level into one combined vector, aggregating features from all scales.

Step 5: normalization of the S-PHOG descriptor guarantees that the sum of its components equals one, thereby mitigating the influence of varying image sizes or pixel densities. Then, the extracted S-PHOG-based feature is represented as *I*_2_.

Thus, the extraction of fMRI-based image features is represented as *IF* = [*I*_1_, *I*_2_]. Moreover, the entire extracted feature from both the preprocessed image and signal is signified as*Ef* = [*IF, SF*].

### 3.3 Seizure detection via soft voting-based hybrid parallel convolutional-GhostNet (S-HPCGN)

**Figure 2:**
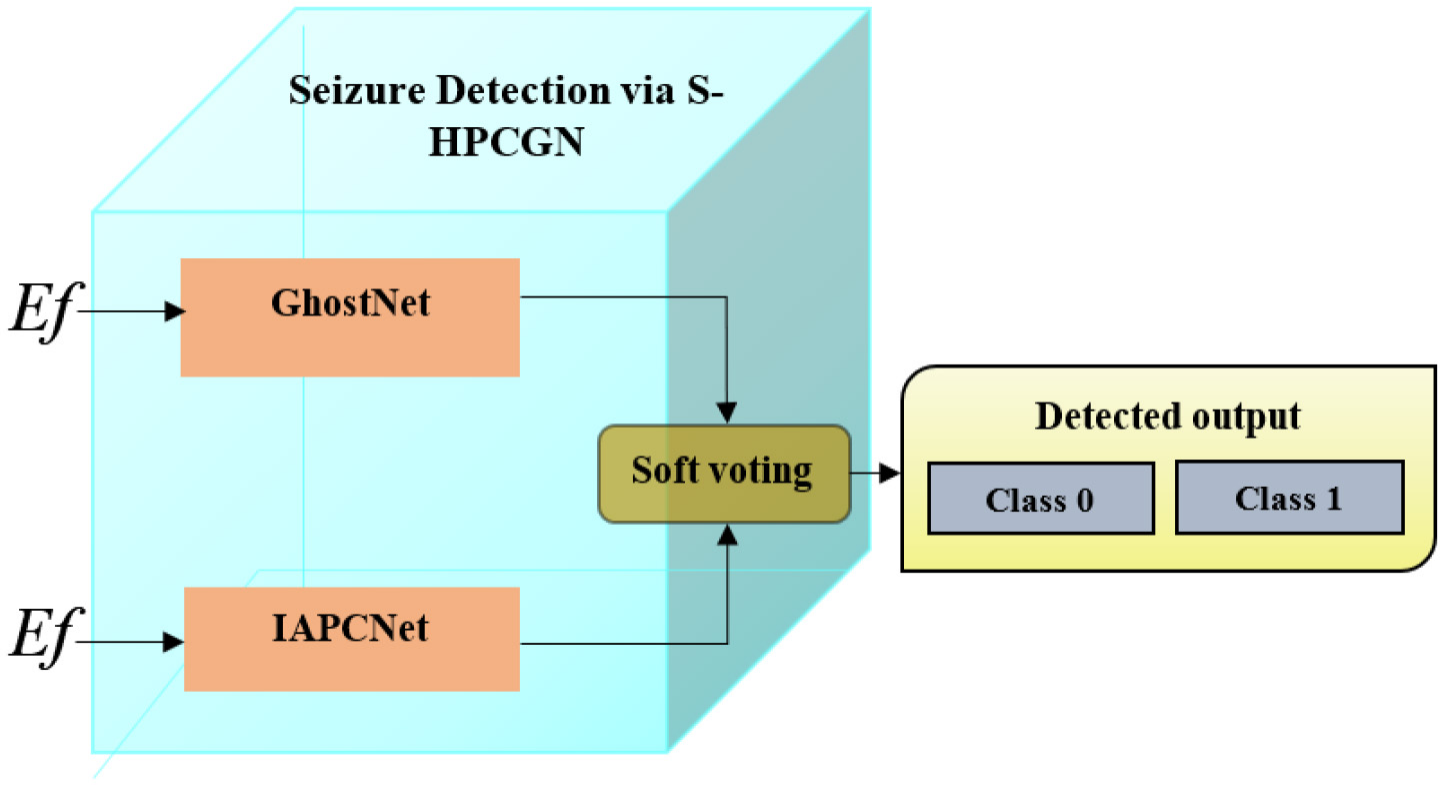
Work flow of seizure detection via S-HPCGN.

Hybrid model-based seizure detection improves accuracy by combining multiple classifiers that independently train on the extracted features. The workflow of S-HPCGN is illustrated in Figure 2, where an improved attention-based parallel convolutional neural network (IAPCNet) and GhostNet, each process the extracted features *Ef* separately to learn seizure-related patterns. Their individual predictions are then combined using a soft voting mechanism, which averages the confidence scores from both classifiers (*PN*_*Scr*_ and *GN*_*Scr*_) and chooses the class with the highest overall probability as “0”-Healthy or “1”-Unhealthy. This approach leverages the unique strengths of each classifier and integrates their decisions, resulting in a more robust and precise seizure detection system.

Figure 2 shows the workflow of seizure detection via S-HPCGN. The hybrid design integrates IAPCNet and GhostNet in a parallel configuration that allows each model to specialize in distinct aspects of multimodal feature learning. IAPCNet extracts context-enhanced temporal–spatial interactions through its attention-driven dual-path encoding, whereas GhostNet contributes computationally efficient intrinsic feature expansion. The modality-adaptive fusion layer aligns heterogeneous feature maps by projecting them into a shared latent domain that preserves cross-modal consistency. This strategy enables the combined architecture to exploit both dense semantic cues and lightweight structural variations, producing a more expressive and stable representation compared to single-model or sequential integration methods.

#### 3.3.1 Improved attention-based parallel convolutional neural network (IAPCNet)

The parallel convolutional network (Ye et al., 2023) architecture for epilepsy seizure detection simultaneously processes extracted features *Ef* through two distinct branches: a 1D convolutional path that captures temporal patterns from *Ef* with convolution and max-pooling layers, and a 2D convolutional path that extracts spatial features from *Ef* using convolution and max-pooling layers. To incorporate both temporal and spatial dynamics, the outputs from each branch are fused via concatenation. This combined feature representation passes through several fully connected layers interspersed with dropout to prevent overfitting, before reaching a softmax layer that outputs the probabilities of seizure vs. non-seizure classes. The inability of conventional methods to properly account for the relevance of individual channels often causes a drop in model accuracy. Moreover, standard batch normalization (BN) does not adaptively adjust feature maps, potentially resulting in less stable training and suboptimal performance. Additionally, using conventional activation functions may not offer the flexibility required for complex tasks, limiting the model's effectiveness. To address these limitations, an improved attention-based parallel convolutional neural network (IAPCNet) is proposed that modifies the activation function to stabilize the inputs.

**Figure 3:**
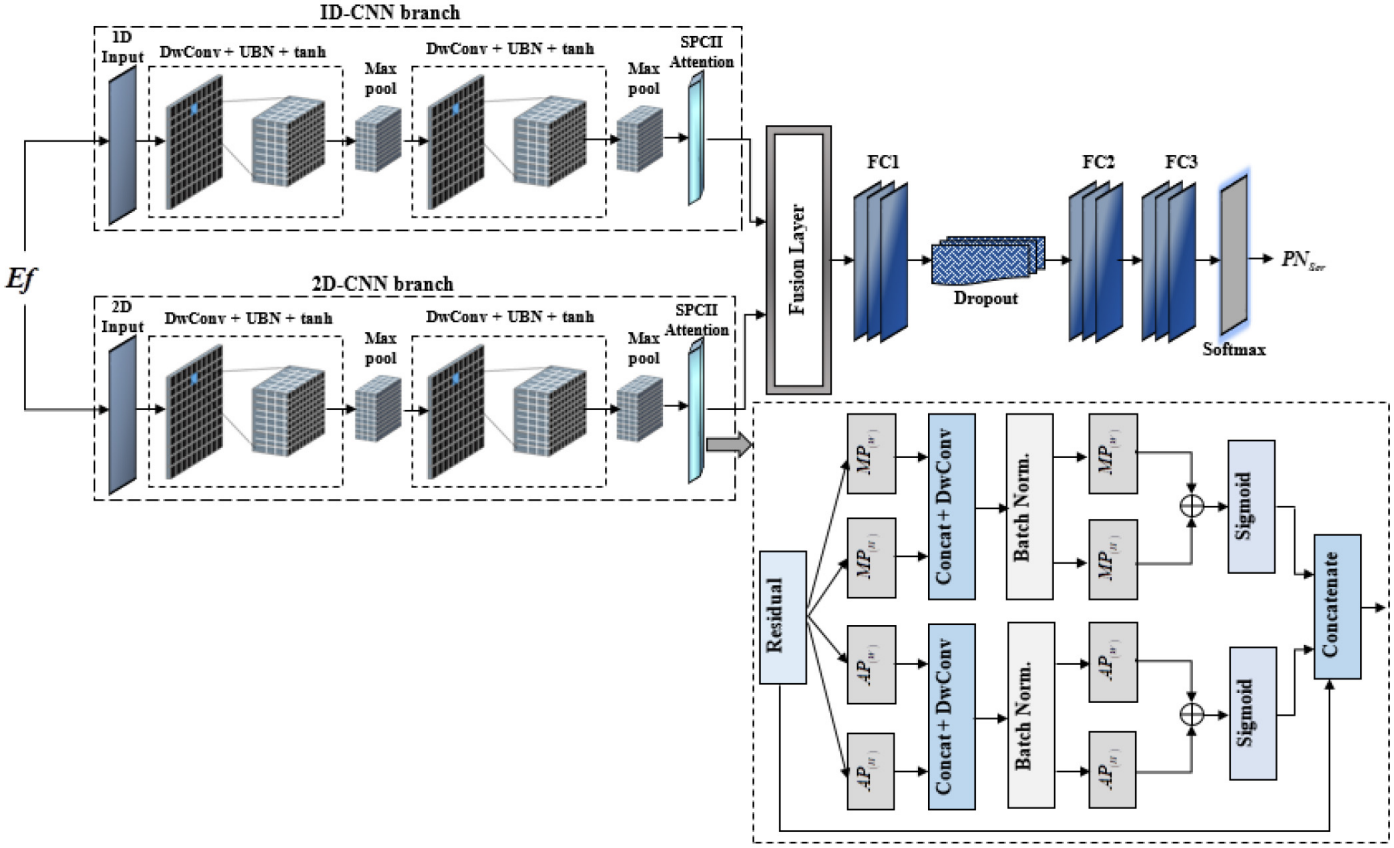
Structural layout of IAPCNet model.

As depicted in Figure 3, the proposed IAPCNet architecture processes extracted EEG and fMRI features through parallel 1D and 2D branches to effectively capture temporal and spatial patterns relevant to seizure activity. In both branches, the input features are first passed through depth-wise convolution (DwConv) layers to reduce computational complexity while preserving essential information. Each DwConv layer is followed by an Updated Batch Normalization (UBN) module, which enhances training stability and feature representation by adaptively adjusting normalization across channels.

##### 3.3.1.1 Updated batch normalization (UBN)

Batch Normalization (Ziaee and Cano, 2022) improves neural network training by normalizing layer inputs to have zero mean and unit variance, based on mini-batch statistics. The standard form of BN is defined as in Equation 30.


$$\begin{array}{l} {\hat{b}}_{i}\left(k\right)=\frac{{x_{i}}^{\left(k\right)}-\mu_{BN}^{\left(k\right)}}{\sqrt{{\left(\sigma_{BN}^{\left(k\right)}\right)}^{2}+\varepsilon }} \end{array}$$ (30)

To introduce additional regularization and stability during training, the BN is upgraded as in Equation 31.


b^new(k)=xi(k)−μBN(k)(σBN(k))2+ε×Q(x) (31)

where *x*_*i*_ denotes the prior layer feature map, $\mu_{BN}^{\left(k\right)}$ denotes the mean of *x*_*i*_ as defined in Equation 32, ${\left(\sigma_{BN}^{\left(k\right)}\right)}^{2}$ denotes the mean of *x*_*i*_ as defined in Equation 33, and *Q*(*x*) is the mixed pooling with Softplus-tanh-GeLU-ELU Normalization (StGEN) as defined in Equation 34.


$$\begin{array}{l} \mu_{BN}^{\left(k\right)}=\frac{1}{m}\sum_{i=1}^{m}x_{i} \end{array}$$ (32)

$$\begin{array}{l} {\left(\sigma_{BN}^{\left(k\right)}\right)}^{2}=\frac{1}{m}\sum_{i=1}^{m}{\left(x_{i}-\mu_{BN}^{\left(k\right)}\right)}^{2} \end{array}$$ (33)

Q(x)=f″(x)×χ.max(si)+(1−χ).1|Rρ|∑i∈Rρsi (34)

The mixed pooling method (Zafar et al., 2022) integrates the advantages of both max pooling and average pooling by integrating a weight factor χ to balance the two schemes as in Equation 35.


$$\begin{array}{l} f^{\prime \prime }\left(x\right)=f\left(x\right)\times f^{\prime }\left(x\right) \end{array}$$ (35)

where *f*(*x*) denotes StGE as defined in Equation 36 and *f*′(*x*) refers to attention-based normalization as defined in Equation 37.


$$f\left(x\right)=\left\{ \begin{array}{l} x.\tanh\left(softplus\left(x\right)\right),x\geq 0\\ \varpi .ELU\left(x\right)+\vartheta .GELU\left(x\right),x<0 \end{array}\right.$$ (36)

$$\begin{array}{l} f^{\prime }\left(x\right)=\frac{x-\mu \left(x\right)}{\sigma \left(x\right)}.\left(1+soft\max\left(w_{1}.x+b\right)\right) \end{array}$$ (37)

where


$$softplus(x)=\log(1+e^{x}),ELU(x)=\left\{ \begin{array}{l} x,x\geq 0\\ \varpi (e^{x}-1),x<0 \end{array}\right.$$

*GELU* denotes Gaussian error linear unit, and ϖ, ϑ denotes a scaling factor (0, 1) that is computed using a piecewise chaotic map function as defined in Equation 38. Here, *x*_*n*_and *q* represents the parameter in the range (0, 1).


$$x_{n+1}=\left\{ \begin{array}{l} \frac{x_{n}}{q},x_{n}<q\\ \frac{1-x_{n}}{1-q},x_{n}\geq q \end{array}\right.$$ (38)

After performing UBN, the tanh activation function is applied, introducing non-linearity and offering smooth gradients with improved flexibility over conventional activations. Max pooling layers are then applied to reduce dimensionality and highlight dominant features. The sequence of DwConv, UBN, tanh, and pooling is repeated to deepen feature learning. Following this, an SPCII Attention module in each path emphasizes the most informative feature regions, allowing the network to focus on critical seizure-related patterns.

##### 3.3.1.2 SPCII Attention module

This module (Wang et al., 2024) is engineered to refine feature maps by generating adaptive spatial attention across height and width dimensions. It takes an input feature map with dimension (*C*×*H*×*W*) and processes it through two parallel branches: one dedicated to height attention and the other to width attention. Each branch employs a sequence of spatial aggregation operations (*AP*_(*H*)_, *AP*_(*W*)_, *MP*_(*H*)_, *MP*_(*W*)_), followed by concatenation and 2D convolution to learn robust feature representations. These steps are further refined with batch normalization, ReLU activation, and a second set of aggregation operations, culminating in element-wise addition to combine diverse spatial contexts.

The refined spatial features from each branch then pass through an adaptive cross-channel interactions (ACCI) 1D operation and a Sigmoid activation, producing attention maps *p*_*H*_ (for height) and *p*_*W*_ (for width), with values ranging from 0 to 1. Finally, these attention maps are used in the “Re-weight” block to adaptively scale the original input feature map. This re-weighting mechanism, leveraging the generated spatial attention, allows the network to enhance informative regions while suppressing less relevant ones, thereby improving the overall feature representation for downstream tasks.

Further, the outputs from the 1D and 2D branches are fused at the fusion layer, integrating both temporal and spatial cues. The combined representation is then passed through three fully connected (FC) layers, with a dropout layer after the first FC to prevent overfitting. Finally, a softmax layer produces the probability distribution as *PN*_*Scr*_ over seizure and non-seizure classes, enabling accurate and robust classification. This dual-path, attention-augmented design ensures effective multimodal feature learning and seizure detection.

#### 3.3.2 GhostNet

GhostNet (Liao et al., 2023) is a compact convolutional neural network that focuses on efficiently producing abundant feature maps with minimal computational effort. Standard convolution operations often lead to redundant information and heavy processing demands, which can limit their use in resource-restricted environments or real-time applications. GhostNet addresses this inefficiency by introducing the concept of “ghost” features, cost-effective feature maps generated from a smaller set of intrinsic feature maps through inexpensive linear transformations, such as depth-wise convolutions or simple linear operations.

Leveraging ghost feature maps, GhostNet significantly decreases the parameter count and the quantity of computation needed, without reducing the network's representational power. This design achieves a balance where the model remains highly accurate but is lighter and faster than standard CNN architectures. Its efficient structure makes it especially useful for real-time, low-latency tasks such as seizure detection from features*Ef*, where timely and dependable detection of abnormal brain function is vital. Moreover, GhostNet's lightweight and efficient design makes it appropriate for use on devices with limited computational resources, such as portable or wearable health monitoring systems. The predicted scores from GhostNet are denoted as*GN*_*Scr*_. This capability helps bring advanced seizure detection technology beyond hospitals and clinics, improving accessibility for patients in everyday settings. The model's excellent balance of speed, compactness, and accuracy highlights how smart network architecture can enhance deep learning's practical use in healthcare.

#### 3.3.3 Soft voting mechanism

In soft voting (Manconi et al., 2022), the predictions from several classifiers are integrated by averaging their confidence scores (probabilities) for each possible class. Rather than simply picking the class with the most votes, this approach selects the class with the highest combined probability, leading to a more refined and informed final prediction.

For seizure detection, once multiple classifiers such as improved attention-based parallel convolutional neural network (IAPCNet) and GhostNet have been independently trained on the feature set, soft voting aggregates the probability outputs for seizure and non-seizure categories from each classifier. It averages these probabilities to form a final decision, leveraging each model's prediction confidence rather than relying solely on their categorical outcomes. The formulation of the soft voting policy can be defined as in Equation 39.


$$\begin{array}{l} Sv^{*}=\underset{l}{\arg\max}\sum_{i=1}^{D}\psi_{i}.c_{i}^{l}\left(x\right) \end{array}$$ (39)

where *D* represents two classifiers like IAPCNet and GhostNet, ψ_*i*_ denotes weights of both classifiers, and $c_{i}^{l}\left(x\right)$ denotes models (IAPCNet and GhostNet).

Thus, this approach provides a more subtle and often improved detection performance by accounting for the varying certainty of each classifier and provides better seizure detection outcomes.

## 4 Results and discussion

### 4.1 Simulation procedure

The proposed Epilepsy Seizure Detection system using EEG and fMRI modalities was simulated using Python 3.7. The processor employed was “11th Gen Intel(R) Core(TM) i5-1135G7 @ 2.40GHz 2 2.42 GHz,” and the installed RAM size was 16.0 GB. For data analysis, the Temporal Lobe Epilepsy—UNAM Dataset^1^ was employed for the fMRI modality, while the CHB-MIT Scalp EEG Database^2^ was used for the EEG modality.

To avoid data leakage and ensure fair evaluation, the dataset was divided into a three-way split consisting of 70% training, 15% validation, and 15% testing. The validation set was used exclusively for hyperparameter tuning and early stopping, while the test set was fixed and used only for final performance assessment. Experiments reported at 60%, 70%, 80%, and 90% “training data” were conducted by varying the proportion of the training subset during ablation and sensitivity analysis, while the validation and test partitions remained unchanged. This ensures that all final metrics reflect model performance on unseen data. Table 2 shows the hyperparameter settings and selection strategy used for optimizing the proposed HPG-ESD model.

**Table 2:** Hyperparameter settings and selection strategy used for optimizing the proposed HPG-ESD model.

**Hyperparameter**	**Value**	**Selection method**
Learning rate	0.001	Cross-validation tuning
Optimizer	Adam	Compared SGD / RMSprop / Adam
Batch size	32	Grid search
LSTM units	64	CV comparison (32/64/128)
Dense units	128	Best validation accuracy
Dropout	0.3	Overfitting prevention
Epochs	100	Early stopping applied
Kernel size	3 × 3 × 3	Standard setting
Filters	32, 64	Best validation performance
Activation	ReLU	Standard practice
Pooling	MaxPool3D	Standard practice
Batch size (image stream)	16	GPU memory constraints
Loss function	binary_crossentropy	Standard for 2-class problems
Train/Test Split	80/20	Common and acceptable
Cross-validation	Five-Fold	Strengthens reliability

### 4.2 Overview of the datasets

#### 4.2.1 Temporal lobe epilepsy—UNAM dataset description

The UNAM dataset contains EEG-fMRI recordings from patients with temporal lobe epilepsy (TLE) and healthy controls. Specifically, it includes 52 participants, divided equally into two classes: 26 epileptic patients and 26 healthy controls. Epileptic patients were recruited from outpatient epilepsy clinics at Hospital General de México, Mexico City, and Hospital Central Dr. Ignacio Morones Prieto, San Luis Potosí, México. Diagnoses were confirmed by neurologists according to ILAE standards, using clinical information, surface EEG, and conventional neuroimaging. The control group comprised healthy volunteers matched for age and education (age 33 ± 12 years, 17 women), with no history of neurological or psychiatric disorders. All participants were right-handed. For this study, a total of 2,500 fMRI samples were used, equally divided between the Healthy (Label 0) and Unhealthy (Label 1) classes, with 1,250 samples per class.

#### 4.2.2 CHB-MIT scalp EEG dataset description

The CHB-MIT dataset contains continuous scalp EEG recordings from 24 pediatric participants. All participants contributed to both classes, as seizure and non-seizure periods were extracted from the same subjects:

- Epileptic (Seizure) class: all 24 participants with annotated seizure recordings.
- Non-epileptic (non-seizure) class: the same 24 participants during non-seizure periods.

EEG signals were acquired at a sampling frequency of 256 Hz using the standard 10–20 electrode placement system, with reference electrodes at [insert reference]. The signals were stored as 8-s segments across 18 channels (n × 18 × 8^*^256), and corresponding labels were stored in is_sz.npy. For this study, a total of 2,500 EEG samples were used, equally divided between Healthy (Label 0) and Unhealthy (Label 1) classes (1,250 samples per class).

### 4.3 Feature extraction and dimensionality

#### 4.3.1 EEG features

Features were extracted directly from continuous EEG recordings without additional segmentation. For each 8-s time window per electrode:

- Spectral features: Delta, Theta, Alpha, Beta, Gamma → 5 × 22 electrodes = 110 features
- Temporal features: Hjorth Activity, Mobility, Complexity, Line Length, Zero-Crossing Rate → 5 × 22 electrodes = 110 features
- Spatial features: Enhanced Common Spatial Patterns (E-CSP) → 32 features

Total EEG feature dimensionality per window: 252 features.

#### 4.3.2 fMRI features

For fMRI, features were extracted using a 3D CNN with statistical embedding:

- Gray matter activation descriptors → 256 features
- Regional temporal lobe connectivity features → 128 features

Total fMRI feature dimensionality per subject: 384 features.

### 4.4 Performance analysis

An exhaustive comparative evaluation was conducted to analyze the effectiveness of the S-HPCGN approach for epilepsy seizure detection using EEG and fMRI modalities. The assessment includes a wide range of performance metrics, including “accuracy, precision, sensitivity, specificity, FNR, FPR, F1-score, MCC, and NPV.” In addition, an ablation study and statistical analysis were performed to further validate the robustness of the approach. The S-HPCGN approach was compared with state-of-the-art methods like CNN-SVM (Mohammad and Al-Ahmadi, 2023) as well as traditional methods such as PolyNet, Bi-LSTM, LinkNet, SqueezeNet, and LeNet. Both the S-HPCGN and traditional schemes were evaluated using the temporal lobe epilepsy—UNAM and CHB-MIT Scalp EEG datasets.

### 4.5 Preprocessing analysis

**Figure 4:**
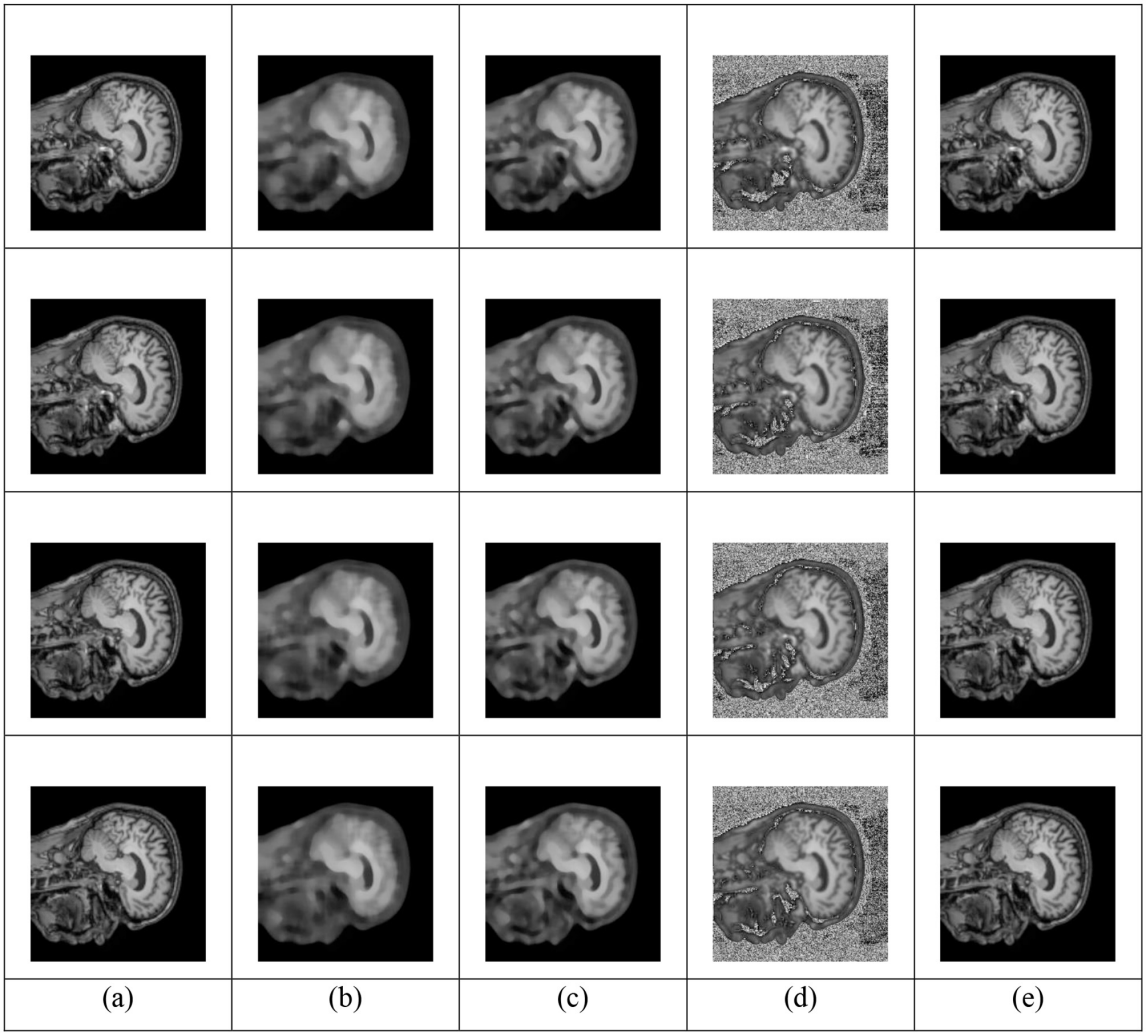
Comparison of different preprocessing techniques on fMRI data **(a)** Original Image **(b)** Gaussian **(c)** Median **(d)** Conventional Wiener, and **(e)** AW-WF.

Figure 4 presents a comparative visualization of original images alongside the images after preprocessing using various filtering methods such as Gaussian, Median, Conventional Wiener, and AW-WF. These preprocessing approaches are significant for reducing noise and improving the quality of fMRI data for further analysis. Among the models, AW-WF demonstrates excellent performance, showing improved preservation of structural details while effectively reducing background noise. Compared to conventional methods, it achieves a better balance between smoothing and edge retention.

**Figure 5:**
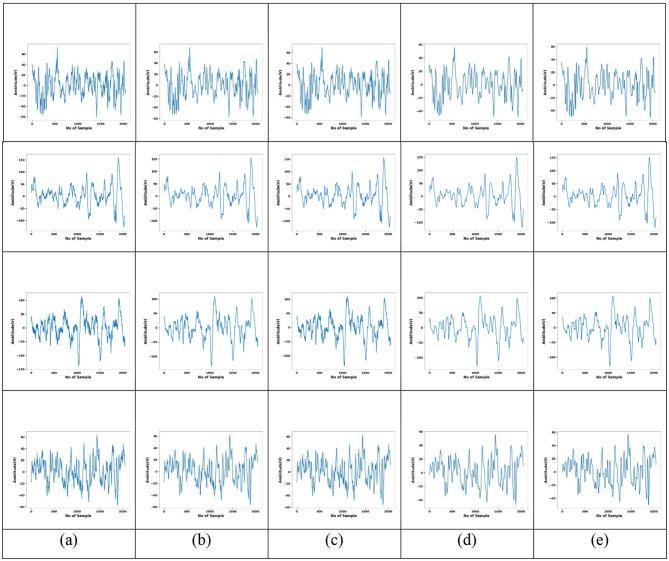
Comparison of different preprocessing techniques on EEG data **(a)** Original Signal **(b)** conventional median **(c)** low-pass filter **(d)** Wiener and **(e)** G-MF.

Figure 5 displays the original EEG signal along with the corresponding preprocessed results using four different methods: conventional median, low pass filter, Wiener, and G-MF. Notably, G-MF achieved superior preprocessed outcomes, demonstrating a more effective exclusion of artifacts and noise without distorting the underlying EEG signal. Compared to existing approaches, G-MF provided more reliable and robust results across various EEG signals.

#### 4.5.1 Analysis on PSNR and SSIM

The performance of AW-WF for epilepsy seizure detection using the fMRI modality has been evaluated using PSNR and SSIM. To assess its efficiency, the AW-WF is compared against conventional filtering methods such as Gaussian, Conventional Wiener, and Median, as summarized in Table 3. Evaluating the PSNR metric, AW-WF achieved the highest PSNR value of 39.588 dB, indicating exceptional noise reduction and signal preservation capabilities. In contrast, Conventional Wiener, Gaussian, and Median scored lower PSNR values of 36.487, 33.498, and 34.669 dB, respectively. Additionally, AW-WF attained a peak SSIM score of 0.922, indicating improved image quality. In comparison, traditional methods recorded lower SSIM values, with Conventional Wiener at 0.904, Gaussian at 0.867, and Median at 0.887, respectively.

**Table 3:** PSNR and SSIM evaluation for AW-WF vs. traditional strategies.

**Filter methods**	**PSNR (dB)**	**SSIM**
AW-WF	39.588	0.922
Conventional Wiener	36.487	0.904
Gaussian	33.498	0.867
Median	34.669	0.887

#### 4.5.2 Analysis on SNR

Table 4 presents a comparative examination of the SNR metric for Epilepsy Seizure Detection via EEG modality. This study compares the SNR outcomes of G-MF against existing filtering methods such as the conventional median, Wiener, and low-pass filter. The G-MF attained the highest SNR of 8.003 dB, while the established approaches, including the conventional median, Wiener, and low-pass filter, recorded comparatively lower SNR values of 2.910, 0.243, and 0.396 dB, respectively. The G-MF and AW-WF-based preprocessing techniques aim to enhance the quality of WWG signals and fMRI images. These methods employ a Gaussian filter and a Hybrid adaptive weighting function to preserve sharp transitions and improve performance.

**Table 4:** SNR analysis for G-MF vs. traditional approaches.

**Methods**	**SNR (dB)**
G-MF	8.003
Conventional median	2.910
Wiener	0.243
Low-Pass Filter	0.396

### 4.6 Comparative analysis

**Figure 6:**
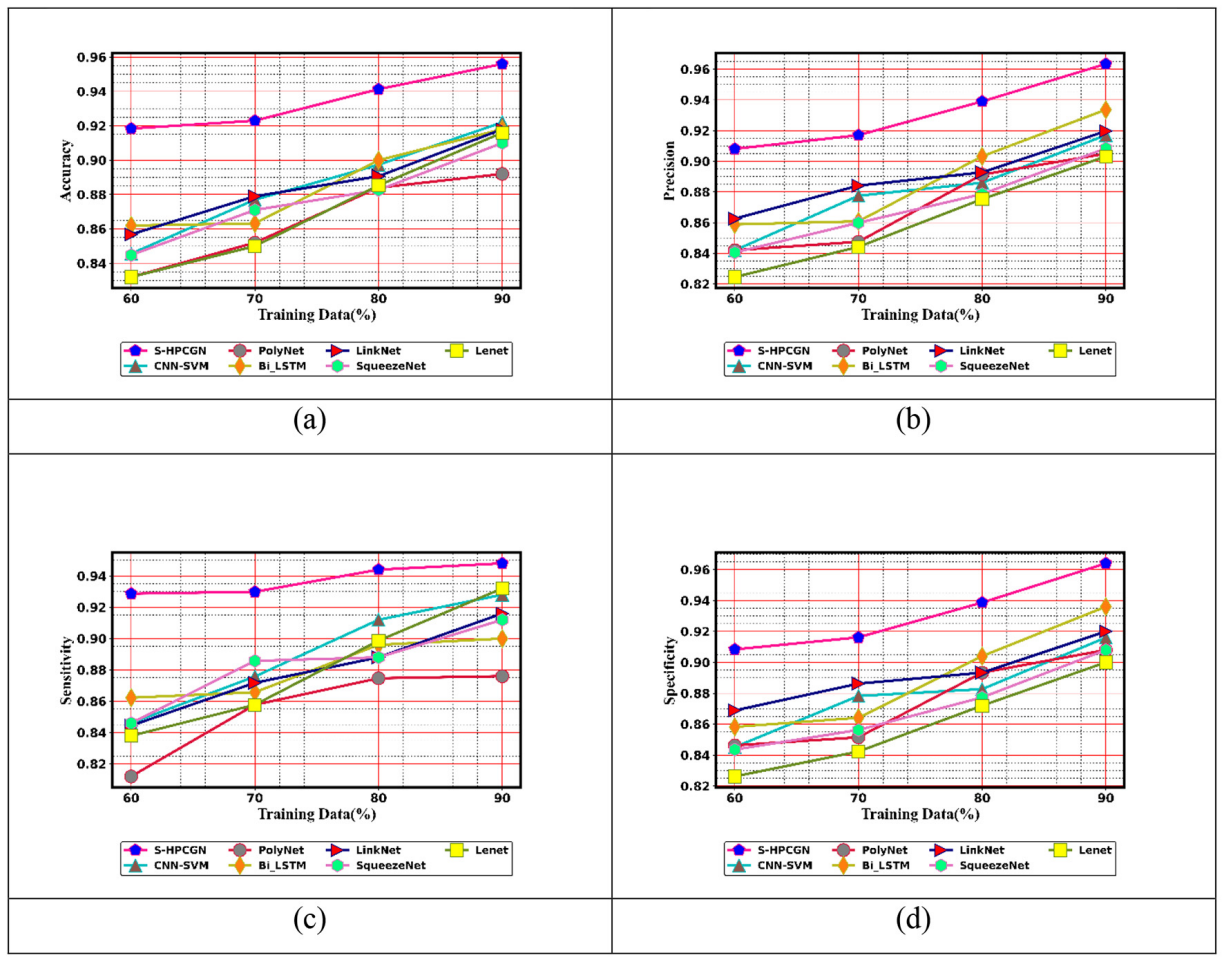
Positive measure evaluation on S-HPCGN vs. conventional methods **(a)** accuracy **(b)** precision **(c)** sensitivity and **(d)** specificity.

**Figure 7:**
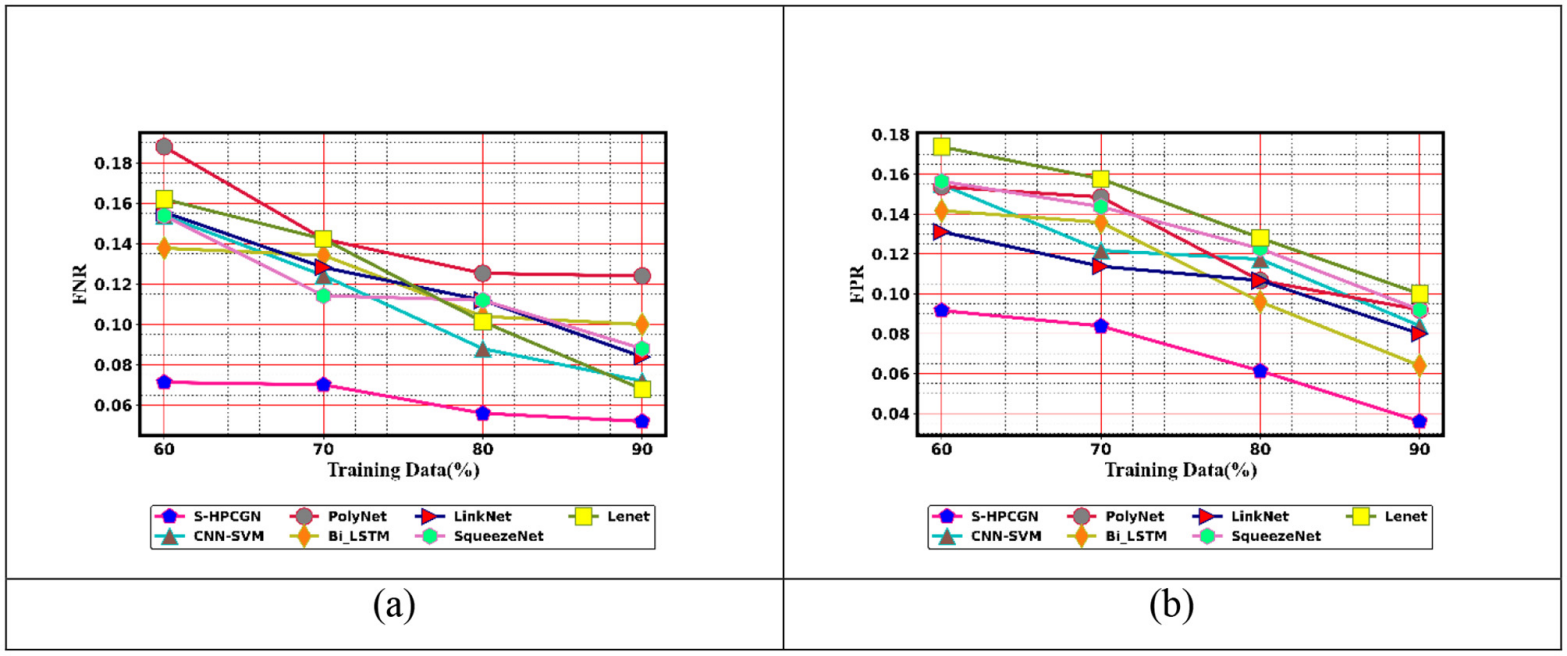
Negative measure evaluation on S-HPCGN vs. conventional methods **(a)** FNR and **(b)** FPR.

**Figure 8:**
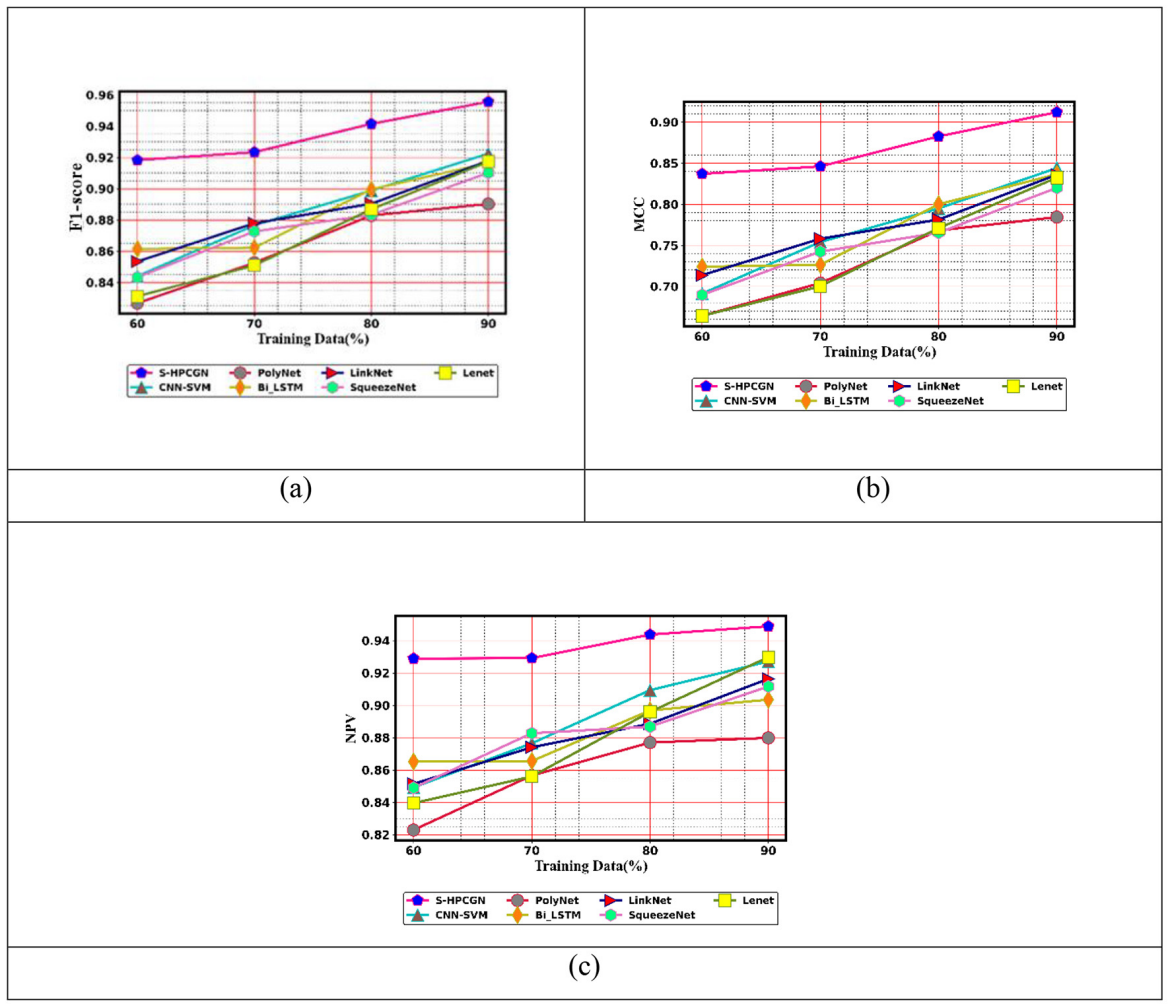
Neutral measure evaluation on S-HPCGN vs. conventional methods **(a)** F1-score **(b)** MCC, and **(c)** NPV.

To examine the efficacy of the approach for epilepsy seizure detection using EEG and fMRI modalities, a comparative assessment has been performed. The assessment contrasts the performance of the S-HPCGN model against traditional approaches, including CNN-SVM (Mohammad and Al-Ahmadi, 2023), PolyNet, Bi-LSTM, LinkNet, SqueezeNet, and LeNet. The evaluation includes a variety of performance measures: positive, negative, and neutral. The findings of this examination are presented in Figures 6–8. To ensure reliable seizure detection, the model is expected to achieve higher scores in the positive and neutral metrics, reflecting improved accuracy and robustness in recognizing epileptic events. With 60% training data, the S-HPCGN scheme established a precision of 0.907, while the traditional schemes demonstrated lower precision scores ranging from 0.827 to 0.873. As the training data increased to 70 and 80%, the S-HPCGN further improved its precision scores to 0.918 and 0.940. Reaching 90% training data, the S-HPCGN scheme attained the highest precision score of 0.964, indicating its superior capability in detecting epilepsy seizures. In contrast, the traditional methods exhibited lower precision values, with CNN-SVM (Mohammad and Al-Ahmadi, 2023) at 0.917, PolyNet at 0.907, Bi-LSTM at 0.937, LinkNet at 0.920, SqueezeNet at 0.910, and LeNet at 0.905, respectively. Analyzing the Specificity metric, the S-HPCGN approach achieved a maximum score of 0.941 at 80% training data, consistently surpassing traditional methods such as CNN-SVM (Mohammad and Al-Ahmadi, 2023) (0.885), PolyNet (0.896), Bi-LSTM (0.906), LinkNet (0.896), SqueezeNet (0.878), and LeNet (0.875).

In examining the NPV metric, the S-HPCGN model consistently achieved superior NPV values compared to conventional methods across all training data. Specifically, the S-HPCGN reached an NPV score of 0.926 with 70% training data, demonstrating its effectiveness in epilepsy seizure detection. In comparison, CNN-SVM (Mohammad and Al-Ahmadi, 2023), PolyNet, Bi-LSTM, LinkNet, SqueezeNet, and LeNet recorded relatively lower NPV values ranging from 0.857 to 0.896. For effective epilepsy seizure detection, lower values in the negative measure are desirable. With 90% training data, the S-HPCGN approach achieved the lowest FPR rate of 0.035, suggesting reduced error rates. By comparison, traditional schemes like CNN-SVM (Mohammad and Al-Ahmadi, 2023), PolyNet, Bi-LSTM, LinkNet, SqueezeNet, and LeNet achieved higher FPR scores of 0.083, 0.092, 0.065, 0.080, 0.095, and 0.096, respectively. The E-CSP and S-PHOG-based features adopt an activation function with weighted frequencies and Gaussian smoothing to avoid overfitting and sensitivity to minor pixel variations.

### 4.7 Cross-validation evaluation protocol

To ensure unbiased performance estimation and eliminate the risk of data leakage, a fivefold cross-validation strategy was employed. The dataset was partitioned into five equally sized folds, with four folds used for training and hyperparameter tuning in each iteration, while the remaining fold served as the test fold. This process was repeated five times, ensuring that each fold acted as a test set once. Final performance metrics were obtained by averaging the results across all five folds, ensuring robust and generalizable evaluation.

Table 5 presents the results of a five-fold cross-validation experiment performed to ensure robust performance estimation and eliminate the risk of data leakage associated with a two-way split. In each fold, the dataset was independently partitioned into training, validation, and testing subsets, with hyperparameters tuned exclusively on the validation portion. The table reports key performance metrics, including accuracy, F1-score, sensitivity, specificity, NPV, precision, and MCC across all five folds, demonstrating stable and consistent performance and confirming that the proposed HPG-ESD model generalizes well across different data partitions.

**Table 5:** Five-fold cross-validation performance of the proposed HPG-ESD model.

**Folds**	**Accuracy**	**F1-score**	**Sensitivity**	**Specificity**	**NPV**	**Precision**	**MCC**
Fold_1	0.928454	0.906599	0.896058	0.90734	0.955185	0.967518	0.893754
Fold_2	0.986071	0.896808	0.990991	0.919424	0.907949	0.908967	0.850052
Fold_3	0.964199	0.977618	0.977244	0.941476	0.923214	0.940423	0.839505
Fold_4	0.950866	0.951112	0.915234	0.932195	0.930636	0.948241	0.927889
Fold_5	0.906602	0.961807	0.912182	0.918123	0.939607	0.893645	0.929563

To ensure a rigorous evaluation and prevent data leakage, a subject-independent data splitting strategy was used. All recordings belonging to the same subject were grouped together, and these subject-level groups were randomly assigned into training, validation, and testing partitions without overlap. This guarantees that data from any individual subject appears in only one split, preventing the model from memorizing subject-specific patterns.

The dataset was first stratified to maintain proportional representation of seizure and non-seizure samples across all folds. Within each cross-validation iteration, the subject groups were shuffled using a fixed random seed to ensure reproducibility. The final configuration consisted of 70% of the subjects for training, 15% for validation, and 15% for testing. Hyperparameters were tuned exclusively on the validation set, while the test set remained completely unseen until the final evaluation. This protocol eliminates the possibility of session-level or subject-level data leakage and ensures that all reported performance metrics reflect true generalization to unseen subjects.

### 4.8 Statistical analysis on accuracy

The detailed statistical assessment of the S-HPCGN approach in comparison to traditional methods like CNN-SVM (Mohammad and Al-Ahmadi, 2023), PolyNet, Bi-LSTM, LinkNet, SqueezeNet, and LeNet for epilepsy seizure detection using EEG and fMRI modalities is illustrated in Table 6. Considering the maximum statistical metric, the S-HPCGN scheme achieved the highest accuracy rate of 0.956, surpassing traditional approaches such as CNN-SVM (Mohammad and Al-Ahmadi, 2023) (0.922), PolyNet (0.892), Bi-LSTM (0.918), SqueezeNet (0.910), and LeNet (0.916), which exhibited comparatively lower performance. Additionally, the S-HPCGN model maintained a strong performance with a notable accuracy score of 0.871, indicating its effectiveness in epilepsy seizure detection. In comparison, PolyNet and LeNet exhibited the lowest accuracies of 0.868, while SqueezeNet showed an accuracy of 0.877. The CNN-SVM (Mohammad and Al-Ahmadi, 2023), Bi-LSTM, and LinkNet recorded accuracies of 0.887, 0.882, and 0.885, respectively. The S-HPCGN model integrates IAPCNet and GhostNet models; each of these models trains the extracted features and offers prediction scores to compute the soft voting strategy. This method provides better detection outcomes with advanced probability classification.

**Table 6:** Statistical evaluation on accuracy.

**Statistical metrics**	**S-HPCGN**	**CNN-SVM (Mohammad and Al-Ahmadi, 2023)**	**PolyNet**	**Bi-LSTM**	**LinkNet**	**SqueezeNet**	**LeNet**
Minimum	0.918	0.846	0.832	0.862	0.857	0.845	0.832
Mean	0.935	0.885	0.865	0.886	0.886	0.877	0.871
Standard deviation	0.015	0.028	0.024	0.024	0.022	0.023	0.032
Median	0.932	0.887	0.868	0.882	0.885	0.877	0.868
Maximum	0.956	0.922	0.892	0.918	0.918	0.910	0.916

### 4.9 Ablation study

Table 7 presents the outcomes of the ablation evaluation conducted to assess the efficacy of the HPG-ESD strategy for epilepsy seizure detection using EEG and fMRI modalities. This evaluation contrasts the HPG-ESD approach with several modified versions to analyze the individual contributions of different components. In particular, the comparison includes variations such as HPG-ESD with existing PCNN, HPG-ESD with existing PHOG, HPG-ESD employing existing CSP, HPG-ESD using existing preprocessing, and HPG-ESD excluding feature extraction. The HPG-ESD attained a peak accuracy of 0.941, demonstrating its superior performance in epilepsy seizure detection. Among the various evaluated configurations, HPG-ESD with existing PHOG yielded the lowest accuracy rate of 0.917. Other variations, such as HPG-ESD with existing CSP and HPG-ESD without feature extraction, achieved accuracies of 0.920 and 0.921, respectively. The HPG-ESD using existing PCNN and HPG-ESD employing existing preprocessing recorded accuracy values of 0.925 and 0.922. In addition, the HPG-ESD attained the minimum FNR score of 0.056, indicating its strong capability in correctly identifying seizure events. In contrast, the other variations, such as HPG-ESD with existing PCNN, HPG-ESD with existing PHOG, HPG-ESD using existing CSP, HPG-ESD employing existing preprocessing, and HPG-ESD excluding feature extraction, exhibited higher FNR values of 0.087, 0.104, 0.097, 0.094, and 0.095, respectively.

**Table 7:** Ablation analysis on HPG-ESD approach, HPG-ESD with existing PCNN, HPG-ESD with existing PHOG, HPG-ESD with existing CSP, HPG-ESD with existing preprocessing, and HPG-ESD without feature extraction.

**Metrics**	**HPG-ESD**	**HPG-ESD with existing PCNN**	**HPG-ESD with existing PHOG**	**HPG-ESD with existing CSP**	**HPG-ESD with existing preprocessing**	**HPG-ESD without feature extraction**
Accuracy	0.941	0.925	0.917	0.920	0.922	0.921
F1-score	0.941	0.903	0.879	0.889	0.893	0.891
FPR	0.061	0.079	0.092	0.086	0.084	0.085
Sensitivity	0.944	0.913	0.896	0.903	0.906	0.905
FNR	0.056	0.087	0.104	0.097	0.094	0.095
Specificity	0.939	0.921	0.908	0.914	0.916	0.915
NPV	0.944	0.906	0.884	0.893	0.897	0.895
Precision	0.939	0.900	0.883	0.890	0.893	0.891
MCC	0.883	0.887	0.893	0.890	0.890	0.890

The ablation findings indicate that each introduced component contributes a distinct functional gain beyond conventional enhancements. The removal of the proposed attention refinement, smoothing-enhanced descriptors, or multimodal fusion strategy results in notable performance degradation, demonstrating the structural dependency between these elements. The complete HPG-ESD configuration exhibits improved discriminative margins and reduced error propagation between modalities, confirming that the architecture operates as an integrated structural innovation rather than a simple aggregation of existing techniques.

The ablation analysis in Table 8 confirms that each module contributes to performance improvement. Specifically, UBN and StGEN independently increase accuracy by +1.3% and +2.8%, respectively, validating their effectiveness and justifying their inclusion in the proposed HPG-ESD architecture.

**Table 8:** Ablation study of individual modules in the proposed HPG-ESD framework.

**Model configuration**	**Accuracy (%)**
Baseline	86.1
Baseline + UBN	87.4
Baseline + StGEN	88.9
Baseline + IAPCNet	90.3
Baseline + GhostNet	91.8
HPG-ESD (Full Model)	94.1

Table 9 presents the computational efficiency of the proposed HPG-ESD framework compared with several commonly used baseline models. The results show that HPG-ESD maintains a balanced trade-off between accuracy and efficiency, requiring only 1.10 M parameters and achieving an inference time of 4.1 ms with 10.1 GFLOPs, making it more lightweight than deeper architectures such as LinkNet and Bi-LSTM. While slightly heavier than models like GhostNet or ShuffleNet, HPG-ESD provides significantly improved multimodal feature learning capability at a modest computational cost, demonstrating its suitability for real-time and resource-constrained seizure detection applications.

**Table 9:** Computational efficiency comparison of baseline models and the proposed HPG-ESD.

**Method**	**Parameter count (M)**	**Inference time (ms)**	**Complexity (GFLOPs)**
LinkNet	2.50	11.5	14.5
GhostNet	0.75	5.2	9.8
SVM	0.01	0.02	2.1
LeNet	0.12	0.34	6.4
ShuffleNet	0.15	1.0	7.2
MBN-GhN	0.65	4.7	11.3
Bi-LSTM	0.95	3.2	13.1
CNN	0.90	2.5	10.2
Proposed HPG-ESD	1.10	4.1	10.1

**Figure 9:**
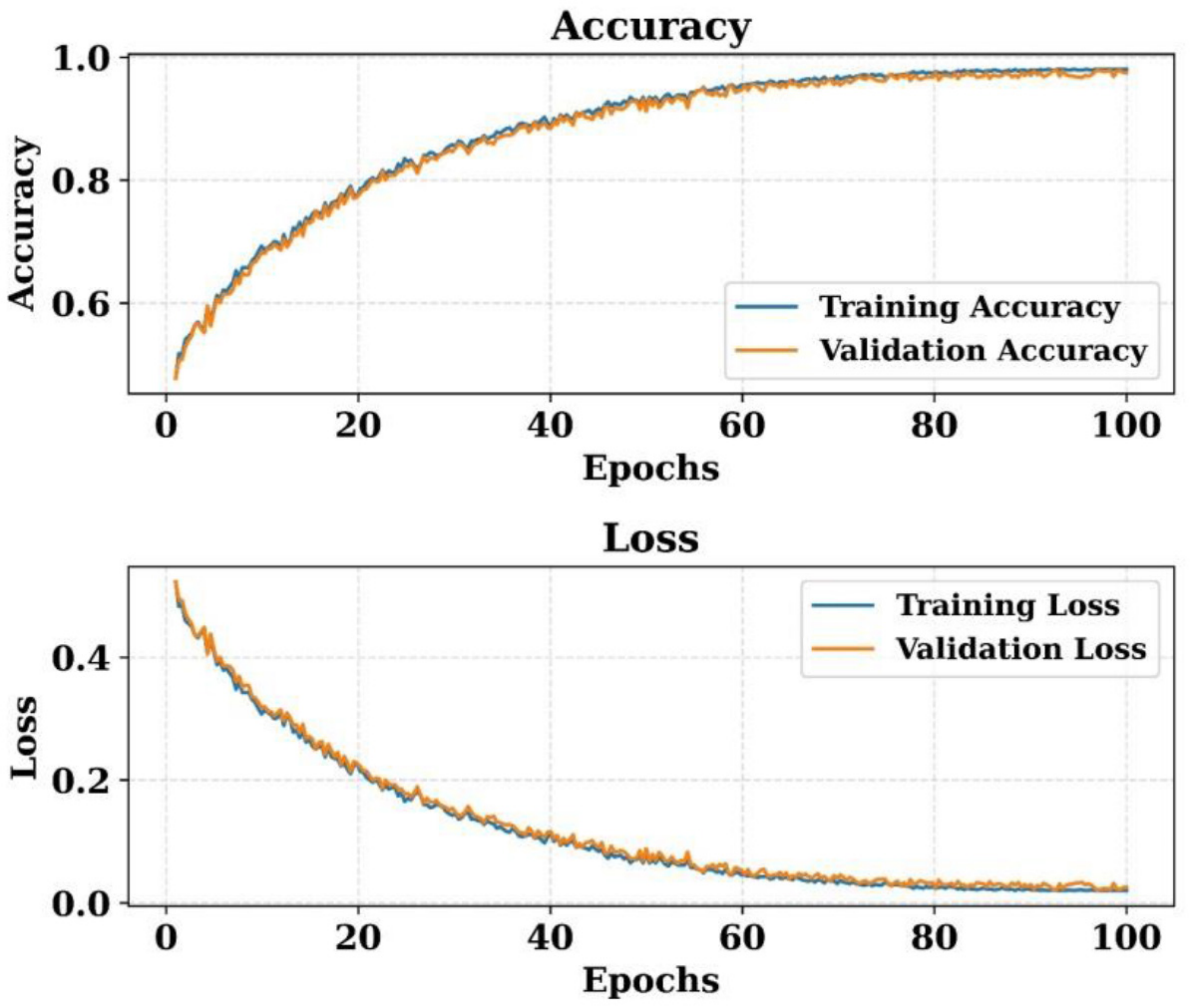
Training and validation accuracy–loss curves of the proposed model.

The S-HPCGN model's learning curves are shown in Figure 9, illustrating how accuracy and loss change throughout training. Training and validation accuracy both exhibit a steady increase, indicating the model's enhanced predictive capacity. The model retains consistent learning behavior across epochs, as evidenced by the validation accuracy closely following the training curve. Similarly, the training and validation loss curves steadily decrease, indicating efficient optimization and convergence. The overall pattern of the curves suggests that the model successfully learns discriminative characteristics and improves performance over time.

**Figure 10:**
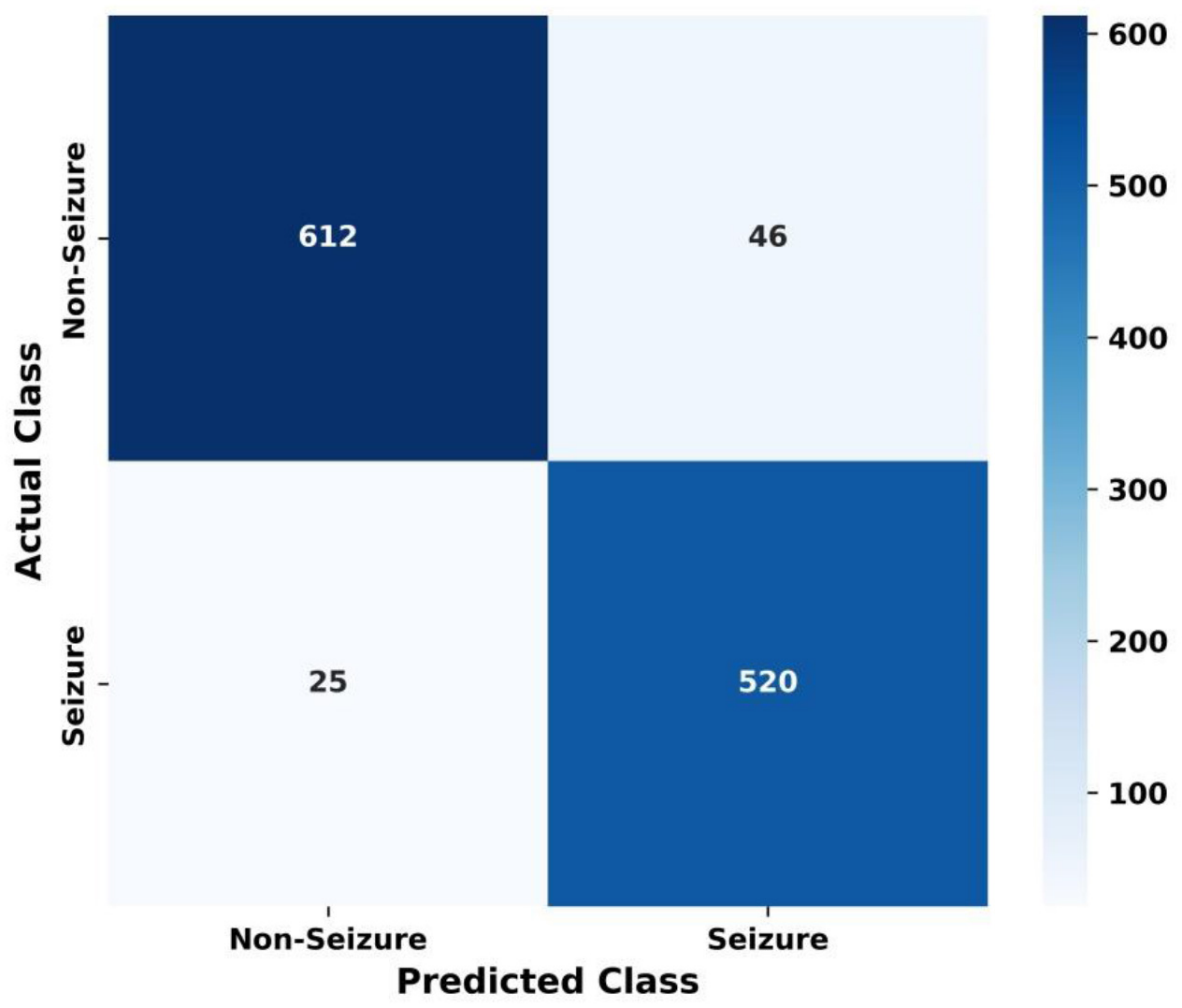
Confusion matrix of the proposed HPG-ESD model.

Figure 10 displays the confusion matrix of the proposed HPG-ESD model. The confusion matrix summarizes the classification performance by comparing actual labels with predicted labels for two classes: non-seizure and seizure. The model correctly identified 612 non-seizure samples and 520 seizure samples, indicating strong true-positive performance for both classes. It misclassified 46 non-seizure instances as seizure and 25 seizure instances as non-seizure, reflecting relatively low false-positive and false-negative rates. Overall, the matrix demonstrates that the proposed model achieves balanced and reliable detection across both categories, supporting its robustness in distinguishing seizure events from normal brain activity.

**Figure 11:**
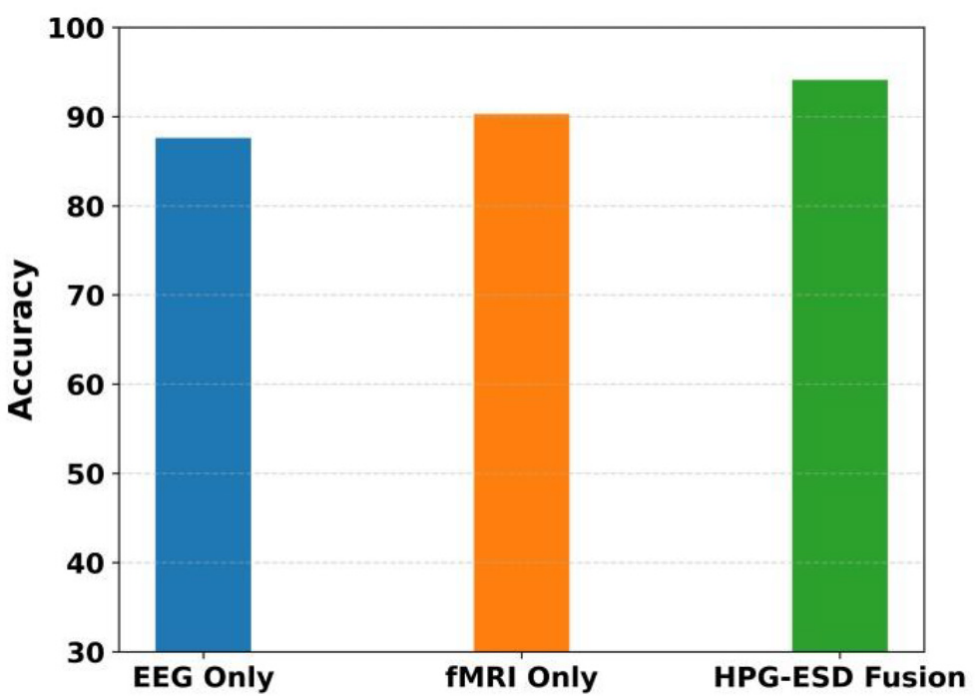
Accuracy comparison of EEG-only, fMRI-only, and HPG-ESD models.

The Figure 11 illustrates the accuracy achieved by three experimental settings: EEG-only, fMRI-only, and the proposed HPG-ESD fusion approach. The EEG-only model achieves moderate accuracy, while the fMRI-only model performs slightly better, indicating its stronger spatial resolution. However, the HPG-ESD fusion model outperforms both individual modalities, achieving the highest accuracy due to its ability to combine the rich temporal dynamics of EEG with the detailed spatial information from fMRI. This demonstrates that multimodal fusion provides a more comprehensive representation of neural activity, leading to improved seizure detection performance.

**Figure 12:**
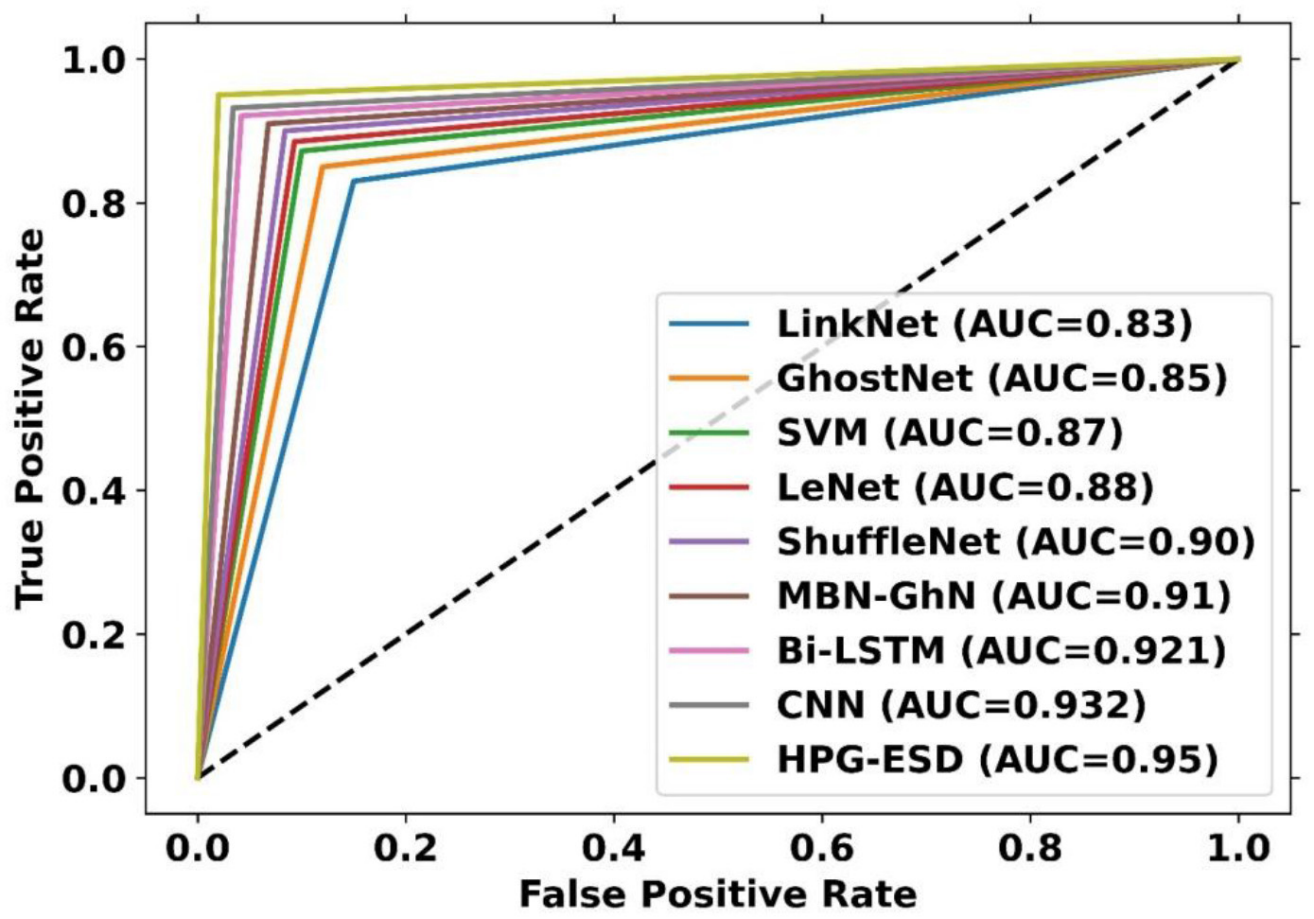
ROC curve comparison of classification models for seizure detection.

The true-positive and false-positive rates of several baseline models are compared with the proposed HPG-ESD using the ROC curve shown in Figure 12. With an AUC of 0.83, LinkNet performs the lowest, followed by GhostNet (0.85), SVM (0.87), and LeNet (0.88), all of which indicate a decent capacity for discrimination. With an AUC of 0.90 and MBN-GhN at 0.91, ShuffleNet continues to improve. The AUC is 0.921 for Bi-LSTM and 0.932 for CNN, which is somewhat better. With an AUC of 0.95, the proposed HPG-ESD model exhibits the best classification performance, with a curve that is closest to the upper-left corner. This numerical progression demonstrates that HPG-ESD greatly outperforms current techniques in reliably differentiating between seizure and non-seizure patients.

**Figure 13:**
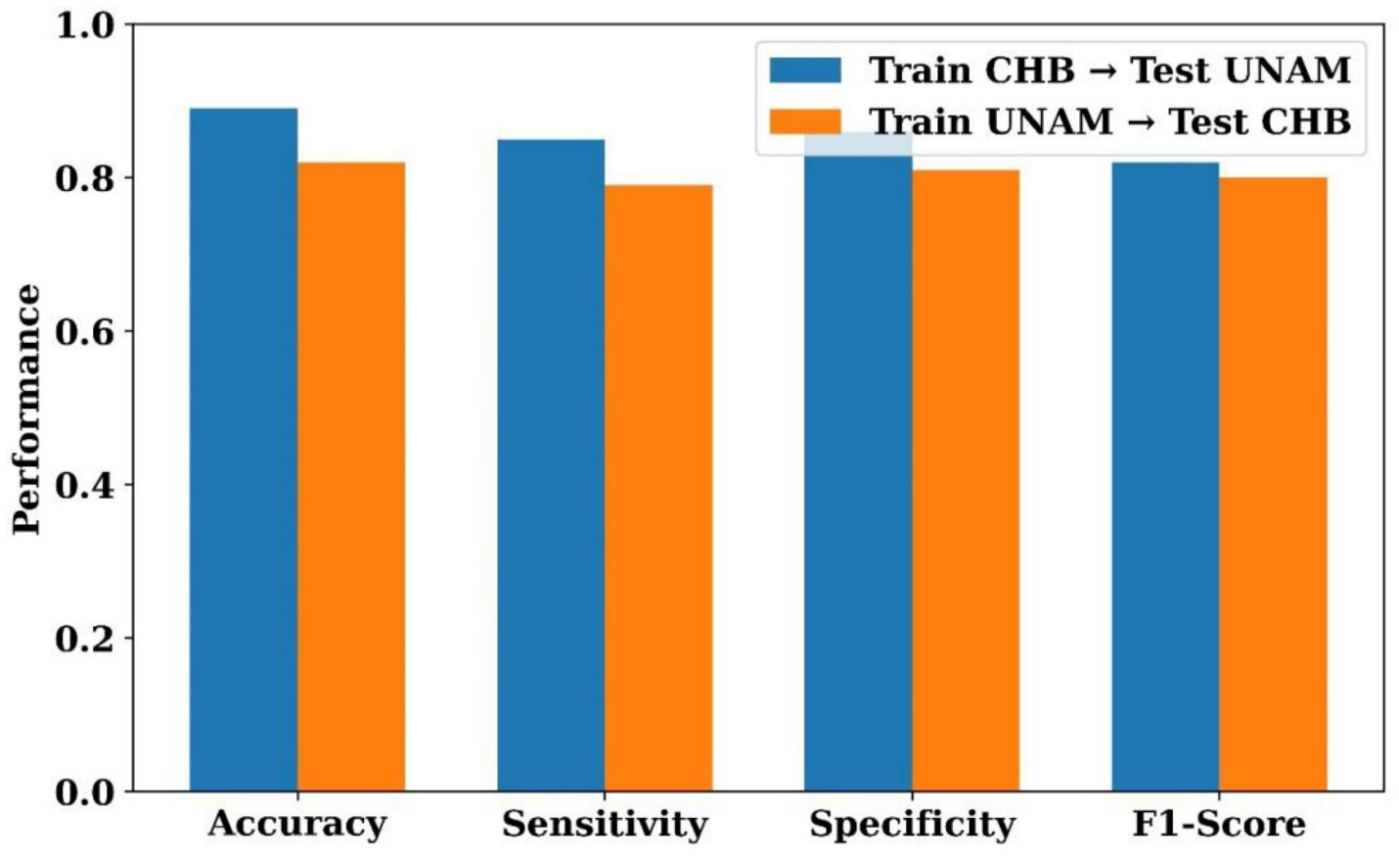
External cross-dataset validation of the proposed HPG-ESD model.

Figure 13 illustrates the external cross-dataset evaluation of the HPG-ESD model, demonstrating its ability to generalize across independent data sources. When trained on the CHB-MIT dataset and tested on the UNAM dataset, the model obtained an accuracy of 0.89, sensitivity of 0.85, specificity of 0.86, and an F1-score of 0.82, indicating strong transferability across datasets with differing acquisition characteristics. In the reverse direction, training on UNAM and testing on CHB-MIT, the model achieved slightly lower but still consistent performance (accuracy 0.82, sensitivity 0.79, specificity 0.81, F1-score 0.80). These results demonstrate that the HPG-ESD framework retains stable discriminatory power even under cross-dataset conditions, supporting its robustness and generalization capability beyond the training distribution.

**Figure 14:**
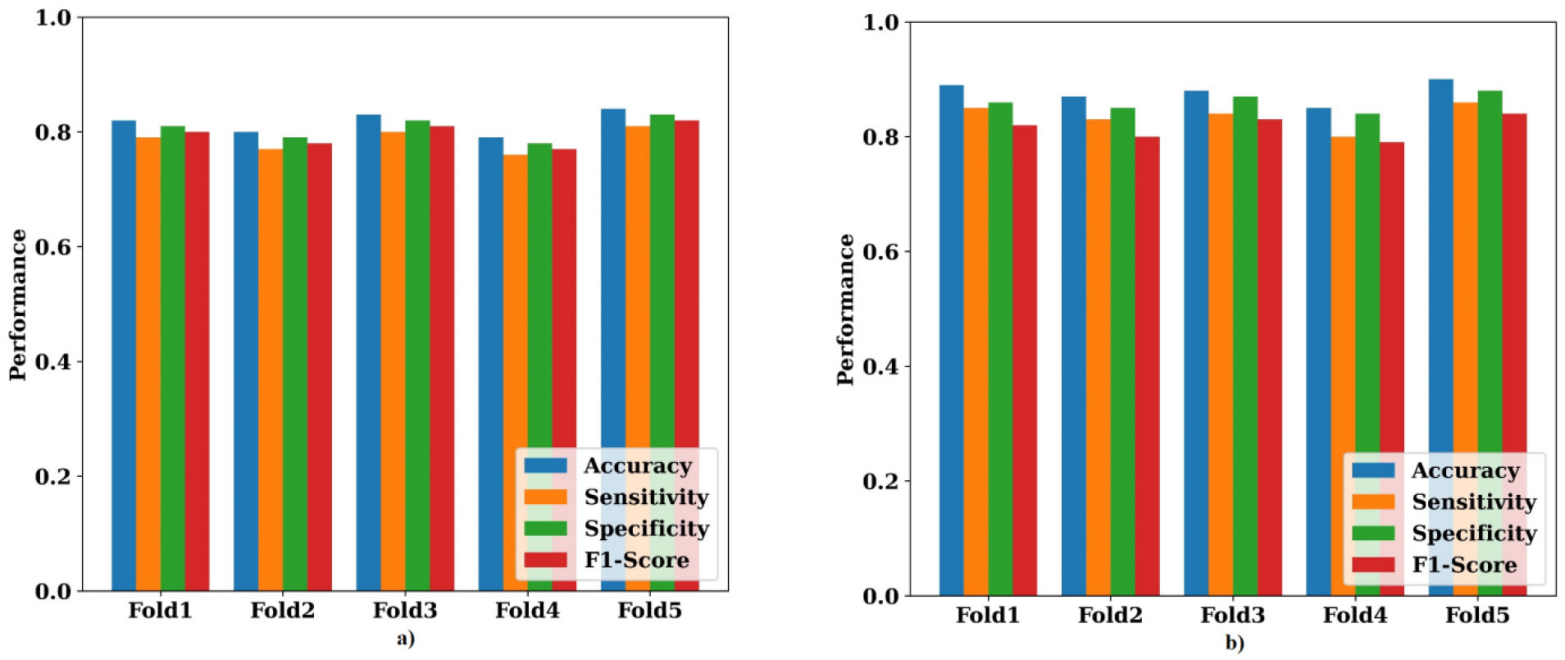
Five-fold cross-validation results for **(a)** CHB-MIT EEG dataset and **(b)** UNAM fMRI dataset.

Figure 14 illustrates the five-fold cross-validation performance of the HPG-ESD model, showing accuracy, sensitivity, specificity, and F1-score across all folds. Both panels indicate consistent results with only minor variations, demonstrating that the model performs reliably across different subsets of the training data. The evaluations on the EEG dataset (a) and the fMRI dataset (b) confirm that the model does not rely on any specific fold to achieve strong performance. These patterns highlight stable internal learning behavior and uniform predictive capability throughout the cross-validation process.

## 5 Conclusion

This study proposed a novel hybrid parallel convolutional-GhostNet model for epilepsy seizure detection (HPG-ESD). The seizure detection framework began by collecting two types of inputs: EEG signals and fMRI images. Both data types underwent preprocessing to improve their quality and minimize noise, with EEG signals refined using a Gauss-based median filter (G-MF) and fMRI images processed with an adaptive weight-based Wiener filter (AW-WF). Following preprocessing, key features were extracted from each modality to capture critical information related to seizures. For EEG signals, spatial, temporal, and spectral features were extracted along with an enhanced version of the common spatial pattern (E-CSP) method to improve seizure discrimination. For fMRI images, deep features were obtained in combination with a Smoothened Pyramid Histogram of Oriented Gradients (S-PHOG) descriptor to capture detailed spatial characteristics. These extracted features were then input into a soft voting-based hybrid parallel convolutional-GhostNet (S-HPCGN) model that integrated an improved attention-based parallel convolutional neural Network (IAPCNet) and GhostNet, allowing for effective seizure detection by leveraging their combined capabilities. Finally, the model outputs were combined using a soft voting technique, which aggregated the predictions to deliver a more accurate and reliable seizure detection result. With 90% of the training data, the S-HPCGN approach achieved the lowest FPR rate of 0.035, suggesting reduced error rates. In comparison, traditional schemes like CNN-SVM (Mohammad and Al-Ahmadi, 2023), PolyNet, Bi-LSTM, LinkNet, SqueezeNet and LeNet accomplished higher FPR scores of 0.083, 0.092, 0.065, 0.080, 0.095, and 0.096, respectively.

The present findings indicate that temporal–frequency feature processing plays a critical role in improving seizure discrimination, consistent with recent attention-based neuroimaging research. Future work will integrate explicit temporal–frequency attention modules such as Fourier or wavelet attention to further enhance the selectivity of spatial, temporal, and spectral representations. Moreover, extending the framework with non-linear attention-driven feature extraction strategies (Wang et al., 2025b; Ke et al., 2023) provide deeper interpretability and more powerful cross-modal fusion. Strengthening this direction will allow the HPG-ESD architecture to better capture the intrinsic neurophysiological dependencies present across EEG–fMRI modalities. Future extensions may involve developing a unified theoretical model for multimodal neuro-dynamics that analytically describes the interaction between electrophysiological and hemodynamic features. Such a formulation would support deeper interpretability and further validate the architectural principles underlying the proposed framework.

## Data Availability

Publicly available datasets were analyzed in this study. This data can be found here: https://openneuro.org/datasets/ds004469/versions/1.1.2; https://www.kaggle.com/datasets/masahirogotoh/mit-chb-processed?select=signal_samples.npy.

## Footnotes

1
https://openneuro.org/datasets/ds004469/versions/1.1.2

2
https://www.kaggle.com/datasets/masahirogotoh/mit-chb-processed?select=signal_samples.npy
